# Myosin-X and talin modulate integrin activity at filopodia tips

**DOI:** 10.1016/j.celrep.2021.109716

**Published:** 2021-09-14

**Authors:** Mitro Miihkinen, Max L.B. Grönloh, Ana Popović, Helena Vihinen, Eija Jokitalo, Benjamin T. Goult, Johanna Ivaska, Guillaume Jacquemet

**Affiliations:** 1 Turku Bioscience Centre, University of Turku and Åbo Akademi University, 20520 Turku, Finland; 2 Faculty of Science and Engineering, Cell Biology, Åbo Akademi University, 20520 Turku, Finland; 3 Electron Microscopy Unit, Institute of Biotechnology, University of Helsinki, 00790 Helsinki, Finland; 4 School of Biosciences, University of Kent, Canterbury, CT2 7NJ Kent, UK; 5 Department of Life Technologies, University of Turku, 20520 Turku, Finland; 6 InFLAMES Research Flagship Center, University of Turku, 20520 Turku, Finland; 7 Turku Bioimaging, University of Turku and Åbo Akademi University, 20520 Turku, Finland

**Keywords:** Filopodia, integrin activity, adhesion, MYO10, talin

## Abstract

Filopodia assemble unique integrin-adhesion complexes to sense the extracellular matrix. However, the mechanisms of integrin regulation in filopodia are poorly defined. Here, we report that active integrins accumulate at the tip of myosin-X (MYO10)-positive filopodia, while inactive integrins are uniformly distributed. We identify talin and MYO10 as the principal integrin activators in filopodia. In addition, deletion of MYO10’s FERM domain, or mutation of its β1-integrin-binding residues, reveals MYO10 as facilitating integrin activation, but not transport, in filopodia. However, MYO10’s isolated FERM domain alone cannot activate integrins, potentially because of binding to both integrin tails. Finally, because a chimera construct generated by swapping MYO10-FERM by talin-FERM enables integrin activation in filopodia, our data indicate that an integrin-binding FERM domain coupled to a myosin motor is a core requirement for integrin activation in filopodia. Therefore, we propose a two-step integrin activation model in filopodia: receptor tethering by MYO10 followed by talin-mediated integrin activation.

## Highlights

• Integrin activity is spatially regulated in filopodia
• Integrin activation at filopodia tips is regulated by talin and MYO10
• MYO10 is required to activate (but not transport) integrins at filopodia tips
• We propose a two-step integrin activation model in filopodia

## Graphical abstract

**Figure:**
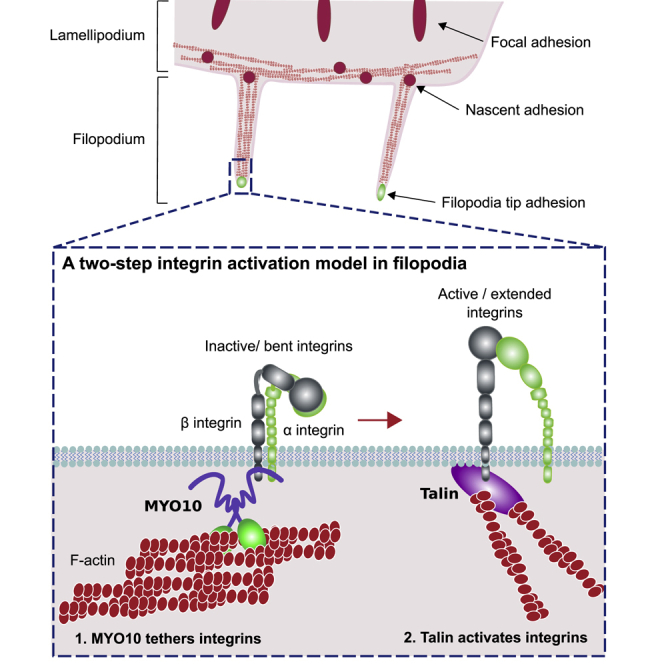

## Teaser

Cells utilize cellular protrusions such as filopodia to explore their environment as they migrate. Filopodia assemble unique integrin-adhesion complexes to sense the extracellular matrix. Miihkinen et al. show that MYO10 and talin regulate integrin activity at filopodia tips and propose a two-step integrin activation model in filopodia.

## Introduction

Filopodia are actin-rich “antenna-like” protrusions that are responsible for constantly probing the cellular environment composed of neighboring cells and the extracellular matrix (ECM). As such, filopodia contain cell-surface receptors, such as integrins, cadherins, and growth factor receptors, that can interact with and interpret a wide variety of extracellular cues (Jacquemet et al., 2015). Filopodia are especially abundant in cells as they migrate in 3D and *in vivo*, where they contribute to efficient directional migration by probing and remodeling the surrounding ECM (Jacquemet et al., 2013, 2017; Paul et al., 2015).

Filopodia have a unique cytoskeleton composed of tightly packed parallel actin filaments with barbed ends oriented toward the filopodium tip (Mattila and Lappalainen, 2008). This organization allows molecular motors, such as unconventional myosin-X (MYO10), to move toward and accumulate at the tips (at approximately 600 nm/s) (Kerber et al., 2009). By doing so, these molecular motors are thought to transport various proteins, including integrins, along actin filaments to the tips of filopodia (Jacquemet et al., 2015; Arjonen et al., 2014; Berg and Cheney, 2002; Hirano et al., 2011; Zhang et al., 2004). In particular, MYO10 is known to bind directly to the NPxY motif of the β-integrin cytoplasmic tail via its FERM (protein 4.1R, ezrin, radixin, moesin) domain (Zhang et al., 2004). At filopodia tips, integrins assemble a specific adhesion complex that tethers filopodia to the ECM (Alieva et al., 2019; Jacquemet et al., 2019; Gallop, 2020). Filopodia adhesions contain several adhesion proteins, including talin, kindlin, and p130Cas, but are devoid of the nascent adhesion markers focal adhesion kinase (FAK) and paxillin (Jacquemet et al., 2019), indicating that filopodia adhesions are distinct in their molecular composition from other adhesion types. The subsequent maturation of these filopodia adhesions into nascent and focal adhesions can promote directional cell migration (Hu et al., 2014; Jacquemet et al., 2016, 2019).

Integrin functions are tightly regulated by a conformational switch that modulates ECM binding, often referred to as activation. Integrin extracellular domain conformations can range from a bent to an extended open conformation, where the integrin’s ligand affinity increases with a stepwise opening (Conway and Jacquemet, 2019; Sun et al., 2019; Askari et al., 2009). For β1-integrin, this unfolding can be viewed using activation-specific antibodies (Byron et al., 2009). Mechanistically, integrin activity can be finely tuned, from within the cell, by multiple proteins that bind to the integrin cytoplasmic tails (Conway and Jacquemet, 2019; Sun et al., 2019; Askari et al., 2009; Bouvard et al., 2013). For instance, talin (TLN), a key integrin activator, can bind to the conserved membrane-proximal NPxY motif of the β-integrin cytoplasmic tail leading to the physical separation of the integrin ɑ and β cytoplasmic tails and integrin activation. Kindlin, another critical regulator of integrin activity, binds to membrane distal conserved NxxY motif in β-integrin cytoplasmic tails, where it cooperates with talin to induce integrin activation (Sun et al., 2019). Although it is clear that integrins and integrin signaling are key regulators of filopodia function (Lagarrigue et al., 2015; Jacquemet et al., 2016, 2019; Gallop, 2020), how integrin activity is regulated within filopodia is not fully understood.

Here, we observed that active (high-affinity) integrin accumulates at filopodia tips, while inactive (unoccupied) integrin localizes throughout filopodia. We find that integrin activation in filopodia is locally regulated by talin and MYO10. Contrary to previous assumptions, the FERM domain of MYO10 is not required to transport integrins to filopodia but instead functions to activate integrins at filopodia tips. Because MYO10 contributes to integrin activation at filopodia tips, but MYO10-FERM alone does not directly activate integrins, our data support a two-step integrin activation model in filopodia. In this model, MYO10 enables integrin receptor tethering at filopodia tips, which is then followed by talin-mediated integrin activation.

## Results

### Integrin activation occurs at filopodia tips independently of cellular forces and focal adhesions

**Figure 1:**
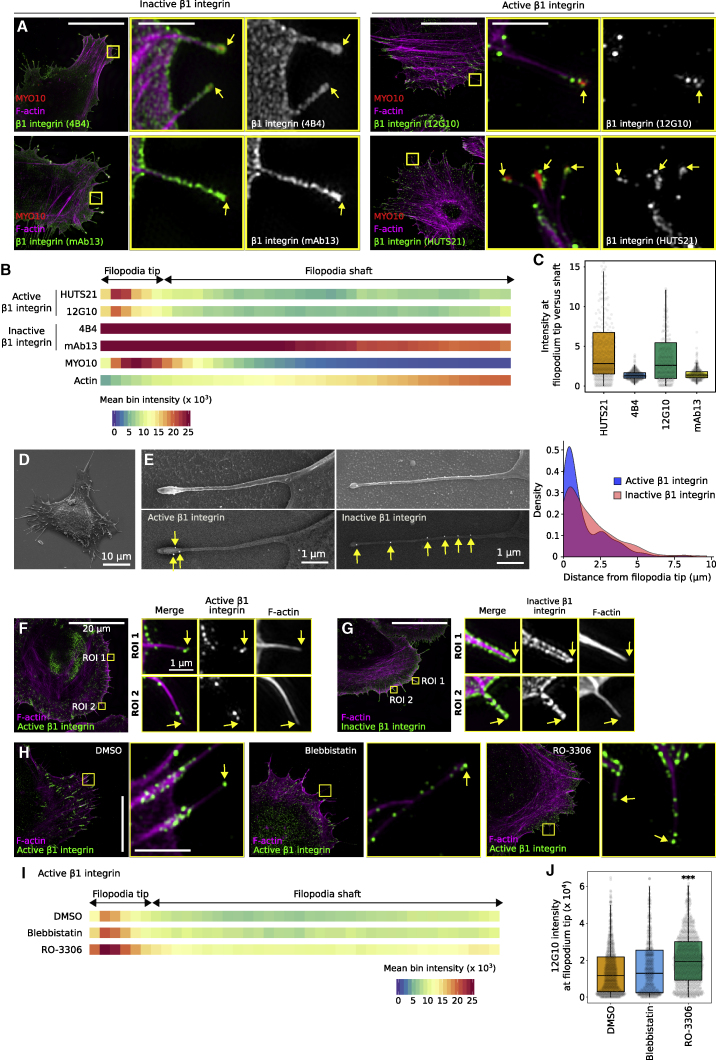
Active integrins accumulate at filopodia tips independently of the cellular forces generated at focal adhesion (A–C) U2-OS cells expressing mScarlet-MYO10 or EGFP-MYO10 were plated on fibronectin (FN) for 2 h, stained for active (12G10 and HUTS21) or inactive (4B4 and mAb13) β1-integrin and F-actin, and imaged using structured illumination microscopy (SIM). Representative maximum intensity projections (MIPs) are displayed; scale bars: (main) 20 μm; (inset) 2 μm. (B) Heatmap highlighting the sub-filopodial localization of the proteins stained in (A) based on their intensity profiles. (C) The preferential recruitment of active and inactive β1-integrin to filopodia tips or shafts was assessed by calculating an enrichment ratio (averaged intensity at filopodium tip versus shaft). Results are displayed as Tukey boxplots. (B and C) MYO10, n = 623 filopodia; F-actin, n = 623; filopodia; HUTS21, n = 538 filopodia; 12G10, n = 329 filopodia; 4B4, n = 413 filopodia; mAb13, n = 369 filopodia; three biological repeats). (D and E) U2-OS cells expressing EGFP-MYO10 were plated on FN for 2 h, stained for active (12G10) or inactive (4B4) β1-integrin, and imaged using a scanning electron microscope (SEM). (E) Representative images of single filopodia are displayed. The upper row was acquired using a secondary electron detector (SED) and the lower row using a backscattered electron detector (vCD). The distance of the two β1-integrin pools from the filopodia tip was measured, and the results are displayed as a density plot (n > 175 gold particles). (F and G) U2-OS cells were plated on FN for 20 min, stained for active (F, 12G10) or inactive (G, 4B4) β1-integrin, and imaged using SIM. Representative MIPs are displayed; scale bars: (main) 20 μm; (inset) 1 μm. (H–J) U2-OS cells expressing EGFP-MYO10 were plated on FN for 1 h and treated for 1 h with 10 μM blebbistatin, 10 μM RO-3306, or DMSO. Cells were stained for active β1-integrin (12G10) and imaged using SIM. (H) Representative MIPs are displayed; scale bars: (main) 20 μm; (inset) 2 μm. (I) Heatmap displaying the sub-filopodial localization of active β1-integrin in cells treated with DMSO, blebbistatin, or RO-3306. (J) The average intensity of 12G10 at filopodia tips measured in (I) are displayed as boxplots (I and J; n > 483 filopodia; three biological repeats; ^∗∗∗^p < 0.001). For all panels, p values were determined using a randomization test. See also Figure S1.

We and others have previously described the formation of integrin-mediated ECM-sensing adhesions at filopodia tips (Shibue et al., 2012; Jacquemet et al., 2019; Lagarrigue et al., 2015; Alieva et al., 2019; Gallop, 2020). To gain further insights into how integrin activity is regulated in MYO10 filopodia, we first assessed the spatial distribution of high-affinity and unoccupied β1-integrin (termed active and inactive integrin, respectively, for simplicity) in U2-OS cells overexpressing fluorescently tagged MYO10 using structured illumination microscopy (SIM) (Figures 1A–1C) and scanning electron microscopy (SEM) (Figure 1D). We focused on β1-integrin because antibodies recognizing the active and inactive forms of this receptor are well characterized (Byron et al., 2009). The average distribution of the β1-integrin species along filopodia was mapped from the SIM and the SEM images revealing enrichment and clustering of active β1-integrins at filopodia tips (Figures 1B–1E). In contrast, inactive β1-integrins were more uniformly distributed along the entire length of the filopodium (Figures 1A–1E). Importantly, this pattern of integrin localization was also recapitulated in endogenous filopodia forming in actively spreading cells (in the absence of MYO10 overexpression) (Figures 1F and 1G).

Previous work reported that forces generated by the actomyosin machinery are required for integrin-mediated adhesion at filopodia tips (Alieva et al., 2019). In addition, we observed that filopodia often align with the force generated by focal adhesions (Stubb et al., 2020). Therefore, we investigated whether cellular forces generated by the cell body and transmitted at focal adhesions were responsible for integrin activation at filopodia tips. U2-OS cells overexpressing fluorescently tagged MYO10 and adhering to fibronectin were treated with DMSO, a myosin II inhibitor (10 μM blebbistatin), or an established focal adhesion inhibitor (CDK1 inhibitor, 10 μM RO-3306) (Robertson et al., 2015; Jones et al., 2018). As expected, inhibition of myosin II or CDK1 led to rapid disassembly of focal adhesions (Figures 1H and S1A). Blebbistatin treatment promoted longer and more numerous filopodia, in line with our earlier report (Stubb et al., 2020), while treatment with the CDK1 inhibitor increased filopodia numbers, but not filopodia length (Figures S1B and S1C). However, no decrease in filopodial integrin activation could be observed when myosin II or CDK1 was inhibited (Figures 1H and 1I). In contrast, CDK1 inhibition led to an increase in the amount of active integrin at filopodia tips (Figures 1J and S1D). Altogether these data indicate that integrin activation at filopodia tips is regulated independently of cellular forces and focal adhesions. Nevertheless, cellular forces are likely required to induce filopodia adhesion maturation into focal adhesions and for efficient ECM sensing (Alieva et al., 2019; Jacquemet et al., 2019).

### Talin is required to activate β1-integrin at filopodia tips

**Figure 2:**
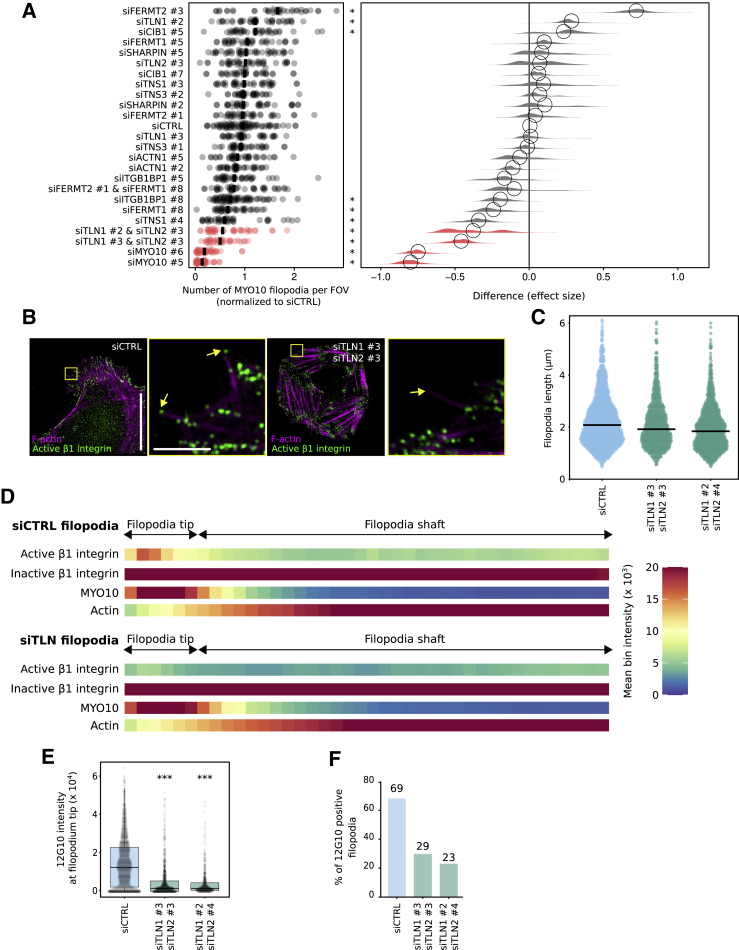
Talin regulates integrin activity at filopodia tips (A) The indicated genes were silenced in U2-OS cells expressing EGFP-MYO10 using siRNA, and the number of filopodia per cell was counted. Results are displayed as dot plots. The effect size was calculated using PlotsOfDifferences (Goedhart, 2019). ^∗^p < 0.05. (B–F) TLN1- and TLN2-silenced U2-OS cells transiently expressing EGFP-MYO10 were plated on FN, stained for active (12G10) or inactive (mAb13) β1-integrin, and imaged using SIM. (B) Representative MIPs are displayed (siTLN1 #3 and siTLN2 #3); scale bars: (main) 20 μm; (inset) 2 μm. (C) Quantification of filopodia length, from SIM images, is displayed as dot plots where the median is highlighted (n > 545 filopodia; three biological repeats). (D) Heatmap highlighting the sub-filopodial localization of the indicated proteins based on their intensity profiles (n > 799 filopodia; three biological repeats, siTLN1 #3 and siTLN2 #3). (E) The average intensity of 12G10 at filopodia tips as measured in (D) is displayed as boxplots (^∗∗∗^p < 0.001). (F) Bar chart highlighting the percentage of filopodia with detectable levels of active β1-integrin in CTRL or siTLN cells (E and F: n > 545 filopodia; three biological repeats). For all panels, p values were determined using a randomization test.

The enrichment of active β1-integrin at filopodia tips (Figure 1) indicates that β1-integrin activation is likely to be spatially regulated by one or multiple components of the filopodium-tip complex. We and others have previously reported that several proteins implicated in the regulation of integrin activity, including the integrin activators talins and kindlins, as well as the integrin inactivator ICAP-1 (ITGB1BP1), accumulate at filopodia tips, where their function remains largely unknown (Lagarrigue et al., 2015; Jacquemet et al., 2016). In addition, we previously reported that enhanced integrin activity often correlates with increased filopodia numbers and stability (Jacquemet et al., 2016). Therefore, we set up a microscopy-based small interfering RNA (siRNA) screen to test the contribution of 10 known integrin activity regulators on filopodia formation. Each target was silenced with two independent siRNA oligos in U2-OS cells stably overexpressing MYO10-GFP (Figure 2A). The effect on MYO10-positive filopodia was scored, and the silencing efficiency of each siRNA was validated by qPCR (Figure S1E) or western blot (Figures S1F and S1G). Of the 10 integrin regulators, only talin (combined TLN1 and TLN2) silencing significantly reduced filopodia numbers. Because kindlin-2 (FERMT2) is a major regulator of integrin activity (Theodosiou et al., 2016) and FERMT2 localizes to filopodia tips (Jacquemet et al., 2019), we were surprised that FERMT2 silencing did not impact filopodia. To validate this further, we imaged filopodia dynamics in cells silenced for both FERMT1 and FERMT2 (over 90% silencing efficiency). There was no effect on filopodia number or dynamics, suggesting that kindlins are not directly required to support filopodia formation or adhesion under the conditions tested (Figures S1H and S1I).

Talin is a critical regulator of integrin activity, known to localize to and modulate filopodia function (Lagarrigue et al., 2015; Jacquemet et al., 2016), and has been predicted by us and others to trigger integrin activation at filopodia tips (Jacquemet et al., 2019; Lagarrigue et al., 2015). To validate this notion, we plated cells silenced for TLN1 and TLN2 on fibronectin and stained for active β1-integrin (Figure 2B). Reduced talin expression did not affect filopodia length (Figure 2C) but was sufficient to decrease active β1-integrin localization at filopodia tips, as well as the percentage of filopodia containing active β1-integrin at their tips (Figures 2D–2F). Altogether, our data demonstrate that talin is required for integrin activation at filopodia tips.

### The FERM domain of MYO10 is required for integrin activation, but not localization, at filopodia tips

We previously observed that FMNL3-induced filopodia rarely contain active β1-integrin (Jacquemet et al., 2019). A careful reanalysis of these data, using intensity profile mapping, indicates that active β1-integrin can be detected in only 23% of FMNL3-induced filopodia (Figures S2A–S2D). However, this is not due to an absence of β1-integrin because all FMNL3-induced filopodia are strongly positive for inactive β1-integrins (Figures S2A–S2D). Because integrin activation is a prominent feature of MYO10-positive filopodia (Figure 1), we hypothesized that MYO10 could functionally contribute to integrin activation in filopodia tips.

**Figure 3:**
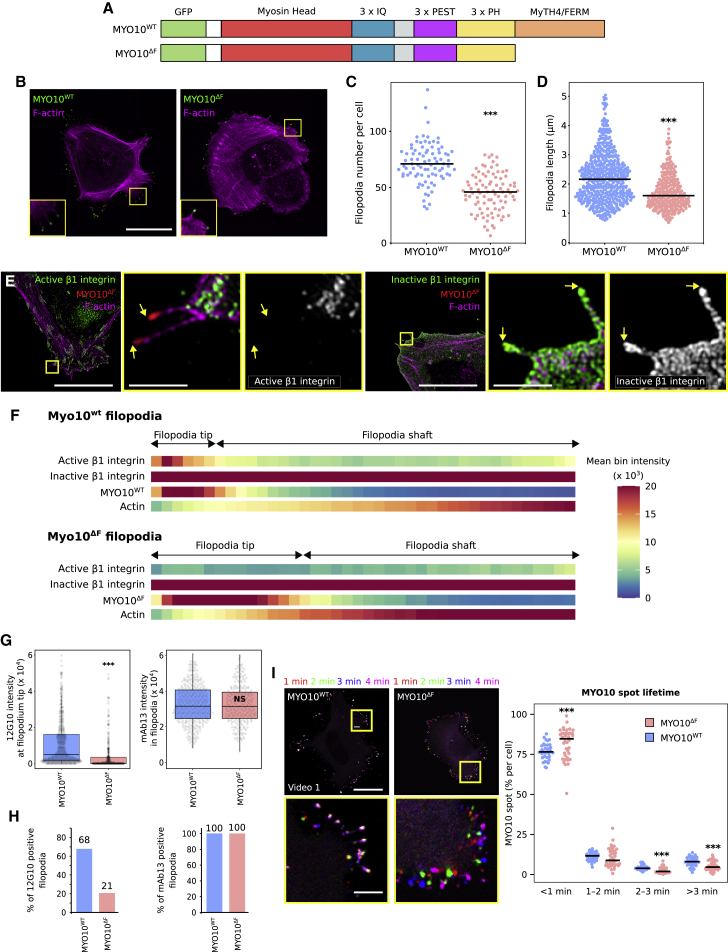
MYO10-FERM is required for integrin activation in filopodia (A) Cartoon of the EGFP-MYO10^WT^ and EGFP-MYO10^ΔF^ constructs. (B and C) U2-OS cells expressing EGFP-MYO10^WT^ or EGFP-MYO10^ΔF^ were plated on FN for 2 h, fixed, and imaged using a spinning-disk microscope. (B) Representative MIPs are displayed. Scale bar: 25 μm. (C) The number of MYO10-positive filopodia per cell was then quantified (n > 85 cells, three biological repeats; ^∗∗∗^p < 0.001). (D) Quantification of MYO10^WT^ and MYO10^ΔF^ filopodia length from SIM images (n > 283 filopodia; three biological repeats; ^∗∗∗^p < 0.001). (E) U2-OS cells expressing EGFP-MYO10^ΔF^ were plated on FN for 2 h, stained for active (12G10) or inactive (mAb13) β1-integrin, and imaged using SIM. Representative MIPs are displayed; scale bars: (main) 20 μm; (inset) 2 μm. (F) Heatmap highlighting the sub-filopodial localization of the proteins stained in (E) generated from their intensity profiles (n > 250 filopodia; three biological repeats). (G) The average intensity of 12G10 at filopodia tips and of mAb13 in filopodia measured in (F) are displayed as boxplots (^∗∗∗^p < 0.001). (H) Bar chart highlighting the percentage of MYO10^WT^ and MYO10^ΔF^-induced filopodia with detectable levels of active (12G10) and inactive (mAb13) β1-integrin (H and G; n > 250 filopodia; three biological repeats). (I) U2-OS cells expressing EGP-MYO10^WT^ or EGFP-MYO10^ΔF^ were plated on FN and imaged live using an Airyscan confocal microscope (scale bar: 25 μm; Video S1). MYO10 spot lifetime is displayed as boxplots (three biological repeats; n > 33 cells; ^∗∗∗^p < 0.006). For all panels, p values were determined using a randomization test. See also Figures S2–S4.

MYO10 directly binds to integrins via its FERM domain (Hirano et al., 2011; Zhang et al., 2004). In this context, MYO10 is thought to transport integrins and other cargo to filopodia tips actively. We assessed the contribution of the MYO10 FERM domain to integrin localization in filopodia by creating a MyTH4/FERM domain deletion construct (MYO10^ΔF^) (Figure 3A). We carefully designed this construct by considering the previously reported MYO10-FERM domain structures (PDB: 3PZD and 3AU5) (Wei et al., 2011; Hirano et al., 2011). MYO10^ΔF^ was overexpressed in U2-OS cells, which express low endogenous MYO10 (Young et al., 2018; Jacquemet et al., 2016). Deleting the MYO10-MyTH4/FERM domain led to a small but significant reduction in filopodia number and filopodia length, in line with previous reports (Zhang et al., 2004; Watanabe et al., 2010) (Figures 3B–3D). Strikingly, the majority of MYO10^ΔF^ filopodia (80%) were devoid of active β1-integrins at their tips (Figures 3E–3H), while the uniform distribution of inactive β1-integrins along the filopodium length remained unaffected (Figures 3E–3H). In line with these results, MYO10^ΔF^-induced filopodia were much more dynamic and seemingly unable to stabilize and attach to the underlying ECM (Figure 3I; Video S1). Taken together, these findings demonstrate that MYO10 and its MyTH4/FERM domain are required for integrin activation at filopodia tips, but not for β1-integrin localization to filopodia tips (Figures 3 and S2).

Video S1. Filopodia dynamics in cells expressing EGP-MYO10WT or EGFP-MYO10ΔF, related to Figure 3 U2-OS cells expressing EGP-MYO10WT or EGFP-MYO10ΔF were plated on fibronectin and imaged live using an Airyscan confocal microscope (1 picture every 5 s over 20 min).

Because these findings challenge the model of the MYO10 MyTH4/FERM domain acting as a cargo transporter of integrin to filopodia tips, we tested whether the presence of inactive β1-integrins in MYO10^ΔF^ filopodia could be because of the low endogenous MYO10 present in these cells. We expressed wild-type (WT) or MYO10^ΔF^ in MYO10-silenced U2-OS cells (90% silencing efficiency with a 3′ UTR-targeting RNA oligo) and analyzed β1-integrin distribution using SIM (Figure S3A). Inactive β1-integrin localization in MYO10^ΔF^ filopodia was not affected by the silencing of endogenous MYO10, further validating that the MYO10 MyTH4/FERM is not required to localize β1-integrin to filopodia (Figures S3B–3E). Interestingly, silencing of endogenous MYO10 led to a small decrease in the percentage of MYO10 filopodia that contain active integrin at their tips, suggesting that integrin activation at filopodia tips by MYO10 may be dose dependent (Figure S3D).

### MYO10-MyTH4/FERM deletion does not influence the localization of established filopodia tip components

Because the MYO10 MyTH4/FERM domain is thought to be the cargo binding site in MYO10 (Wei et al., 2011), we hypothesized that the lack of integrin activation at the tip of MYO10^ΔF^ filopodia would be caused by the absence of a key integrin activity modulator. We co-overexpressed six established filopodia tip components (Jacquemet et al., 2019), TLN1, FERMT2, CRK, DIAPH3, BCAR1, and VASP, with either MYO10^WT^ or MYO10^ΔF^. SIM microscopy revealed that the localization of these proteins was unaffected by MYO10-FERM domain deletion (Figure S4). Interestingly, VASP has been previously described as an MYO10-FERM cargo, but its localization at filopodia tips was unaffected by MYO10-FERM deletion (Young et al., 2018; Tokuo and Ikebe, 2004; Lin et al., 2013). Altogether, our results demonstrate that the recruitment of key filopodia tip proteins, including TLN1, is independent of the MYO10 FERM domain and suggest that MYO10-FERM may regulate integrin activity via another mechanism than cargo transport.

### The interaction between MYO10 and integrins regulates integrin activation at filopodia tips

The MYO10 MyTH4/FERM domain comprises four subdomains, namely, a MyTH4 subdomain and three FERM lobes F1, F2, and F3. To further dissect which part of MYO10-FERM is responsible for mediating integrin activation at filopodia tips, we generated two additional MYO10 deletion constructs where either the F2F3 (MYO10^ΔF2F3^) or the F3 (MYO10^ΔF3^) lobes are missing (Figure S5A). We overexpressed MYO10^ΔF2F3^, MYO10^ΔF3^, MYO10^ΔF^, and MYO10^WT^ in U2-OS cells and compared their filopodia properties (Figures S5B–S5E). MYO10^ΔF2F3^ and MYO10^ΔF3^ filopodia were shorter than MYO10^WT^ filopodia but longer than MYO10^ΔF^ filopodia, indicating that the MyTH4, F1, and F3 subdomains contribute to filopodia elongation (Figure S5C). Importantly, MYO10^ΔF2F3^ and MYO10^ΔF3^ filopodia displayed low amounts of active β1-integrin at their tips, indicating that the MYO10 F3 subdomain is required to activate integrin at filopodia tips (Figures S5D–S5F). These data also indicate that the MyTH4, F1, and F2 subdomains are not directly required to modulate integrin activity at filopodia tips. As others have shown that the MYO10 F3 subdomain contains the β1 integrin binding site (Zhang et al., 2004), our results led us to speculate that MYO10 needs to interact with integrin directly to promote integrin activation.

**Figure 4:**
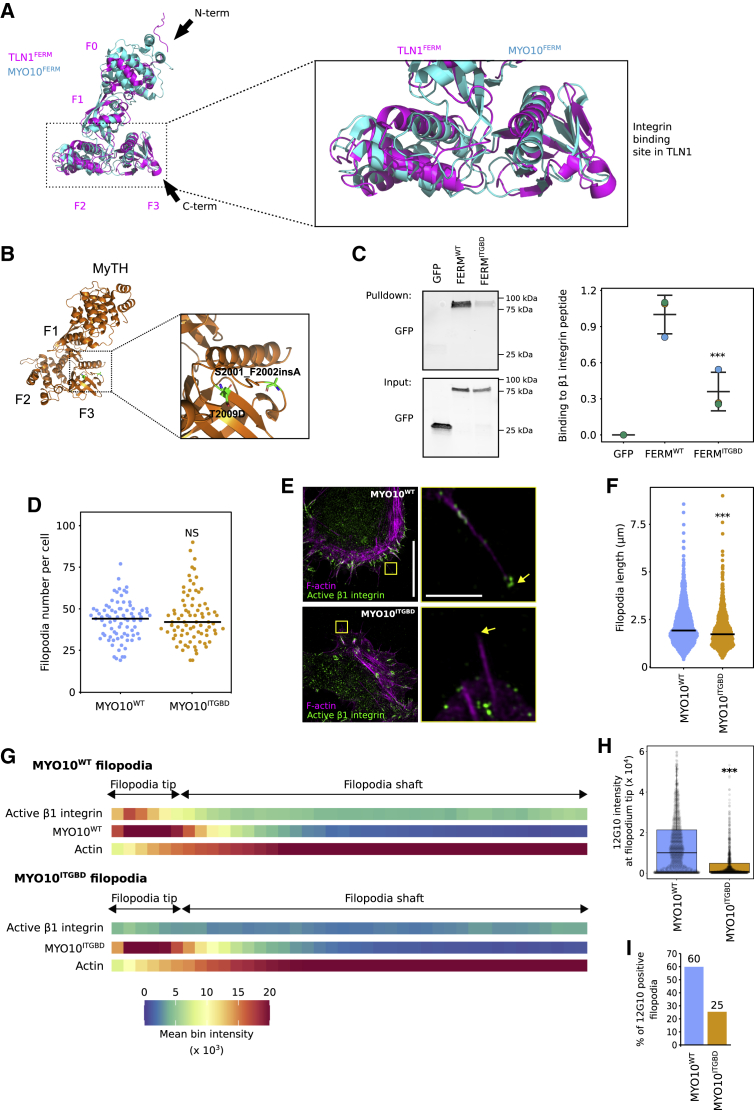
An intact integrin binding site within MYO10-FERM is required for MYO10-mediated integrin activation at filopodia tips (A) Visualization of MYO10-FERM (PDB: 3PZD) (Wei et al., 2011) and TLN1-FERM (PDB: 6VGU) (Zhang et al., 2020) structures. The integrin-binding region on the talin-FERM domain is highlighted and magnified. (B) The structure of the MYO10-FERM mutated on the predicted integrin binding site was modeled using SWISS-MODEL (Waterhouse et al., 2018) based on the MYO10-FERM structure (PDB: 3PZD). (C) β1-Integrin tail peptide pull-down in U2-OS cells expressing EGFP-tagged MYO10-FERM wild-type (WT; FERM^WT^) or mutant (FERM^ITGBD^) or EGFP alone. MYO10-FERM recruitment to the β1-integrin tail was assessed using western blot (n = 3, ^∗∗∗^p = 0.008, Welch’s t test). Individual repeats are color-coded (Lord et al., 2020; Goedhart, 2021). (D) U2-OS cells transiently expressing full-length EGFP-MYO10^WT^ or EGFP-MYO10^ITGBD^ were plated on FN for 2 h, fixed, and imaged using a spinning-disk microscope. The number of MYO10-positive filopodia per cell was quantified (n > 81 cells; three biological repeats). (E–H) U2-OS cells expressing EGFP-MYO10^WT^ or EGFP-MYO10^ITGBD^ were plated on FN for 2 h, stained for active β1-integrin (12G10), and imaged using SIM. (E) Representative MIPs are displayed; scale bars: (main) 20 μm; (inset) 2 μm. (F) Quantification of MYO10^WT^ and MYO10^ITGBD^ filopodia length from SIM images (n > 693 filopodia; three biological repeats; ^∗∗∗^p < 0.001). (G) Heatmap highlighting the sub-filopodial localization of the indicated proteins based on their intensity profiles. (H) The average intensities of 12G10 at filopodia tips measured in (G) are displayed as boxplots (^∗∗∗^p < 0.001). (I) Bar chart highlighting the percentage of MYO10^WT^ and MYO10^ITGBD^ filopodia with detectable levels of active β1-integrin (G–I; n > 693 filopodia; three biological repeats). For all panels except (C), p values were determined using a randomization test. NS, no statistical difference between the mean values of the highlighted condition and the control. See also Figure S5.

Although the site where β1-integrin binds to MYO10-FERM remains unknown, the integrin binding site has been mapped in talin-FERM. Despite some controversy regarding the full talin-FERM structure, superimposition of talin and MYO10 FERM domains revealed that both adopt a similar fold in the β-integrin tail binding subdomains (Figure 4A; Figure S6A) (Zhang et al., 2020; Elliott et al., 2010). Therefore, we can predict mutations likely to disturb the MYO10-integrin interaction (S2001_F2002insA and T2009D; Figure 4B). The introduction of these mutations in MYO10-FERM (FERM^ITGBD^) led to a 64% reduction in the ability of β1-integrin tail peptides to pull down GFP-tagged MYO10-FERM domains from cell lysate, indicating that these mutations can impede the interaction between MYO10 and integrins (Figure 4C). Cells expressing full-length MYO10 with the integrin-binding mutation (MYO10^ITGBD^) generated filopodia to the same extent as cells expressing MYO10^WT^ (Figures 4D and 4E), but MYO10^ITGBD^ filopodia were shorter than MYO10^WT^ filopodia (Figure 4F). Notably, only 25% of MYO10^ITGBD^ filopodia contained detectable levels of active β1-integrin at their tips (Figures 4G–4I). Thus, we conclude that an intact integrin binding site within MYO10-FERM is required for MYO10 to activate β1-integrin at filopodia tips efficiently.

### Unlike Talin-FERM, the MYO10 MyTH4/FERM domain is not able to activate integrins

**Figure 5:**
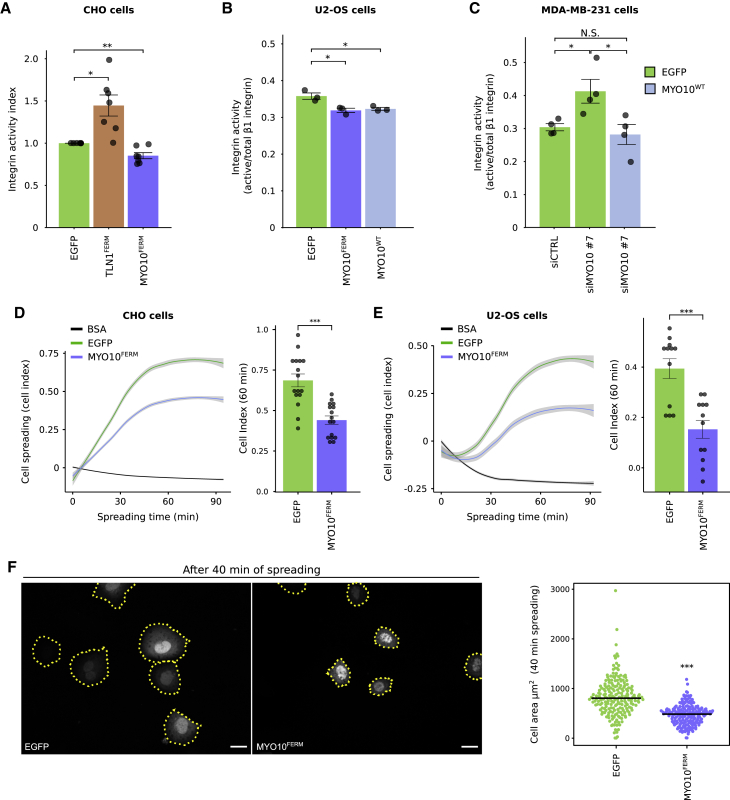
The MYO10 FERM domain inhibits integrin activity (A) CHO cells expressing EGFP, EGFP-TLN1^FERM^, or EGFP-MYO10^FERM^ were either incubated with an Alexa 647-labeled FN fragment (FN7–10) and fixed or fixed directly and stained for ITGA5 (PB1). Samples were then analyzed by flow cytometry, and the integrin activity index was calculated (see STAR Methods; ^∗^p = 0.012, ^∗∗^p = 0.0062, one-sample t test; n = 7 of biological repeats). (B and C) Cells transiently expressing various EGFP constructs (U2-OS) (B) or silenced for MYO10 (siMYO10 #7) and expressing EGFP or EGFP-MYO10 (MDA-MB-231) (C) were fixed and stained for active (9EG7) or total β1-integrin (P5D2). Staining intensity was recorded by flow cytometry, and integrin activation was calculated as a 9EG7/P5D2 ratio (^∗^p < 0.05, Student’s two-tailed t test; B, n = 5 biological repeats; C, n = 4 biological repeats). (D and E) CHO or U2-OS cells transiently expressing EGFP or EGFP-MYO10^FERM^ were left to adhere to FN, and their spreading was monitored over time using the xCELLigence system. The cell index over time is displayed; gray areas indicate the 95% confidence intervals. The cell index at 60 min is also displayed as a bar chart (^∗∗∗^p < 0.001, Student’s two-tailed t test; D, n = 4 biological repeats; E, n = 3 biological repeats). (F) U2-OS cells transiently expressing EGFP or EGFP-MYO10^FERM^ were seeded on FN and allowed to spread for 40 min prior to fixation. Samples were imaged using a confocal microscope and the cell area measured (^∗∗∗^p < 0.001, randomization test; n > 188 cells; 3 biological repeats; scale bars: 16 μm). For all panels, error bars represent the standard error of the mean. See also Figure S6.

The talin-FERM domain is necessary and sufficient to activate integrins (Anthis et al., 2009; Lilja et al., 2017). Given our data indicating that MYO10-FERM is required to activate integrin at filopodia tips (Figures 3 and 4), we tested whether MYO10-FERM could modulate integrin activity similarly to talin-FERM. We employed a flow cytometric assay to measure active cell-surface integrins relative to total cell-surface integrins (Lilja et al., 2017) (Figures 5A–5C). As expected, overexpression of the talin-FERM domain significantly increased integrin activity (Figure 5A). In contrast, overexpression of the MYO10-FERM domain failed to activate integrins and instead led to a small but highly reproducible decrease in integrin activity in CHO and U2-OS cells (Figures 5A and 5B). Similar data were obtained in U2-OS cells overexpressing full-length MYO10 (Figure 5B). Conversely, silencing of MYO10 increased integrin activity in MDA-MB-231 cells, where mutant p53 drives high endogenous MYO10 levels (Arjonen et al., 2014), and this was reversed by the reintroduction of full-length MYO10 (Figure 5C and S6B). Consistent with decreased integrin activation, MYO10-FERM expression attenuated cell adhesion/spreading on fibronectin over time (Figures 5D–5F) (Hamidi et al., 2017). Altogether, our data indicate that, even though the MYO10-FERM domain is necessary for spatially restricted integrin activation at filopodia tips, the MYO10-FERM domain alone cannot activate integrins.

### Unlike Talin-FERM, MYO10-FERM binds to both α- and β-integrin tails

**Figure 6:**
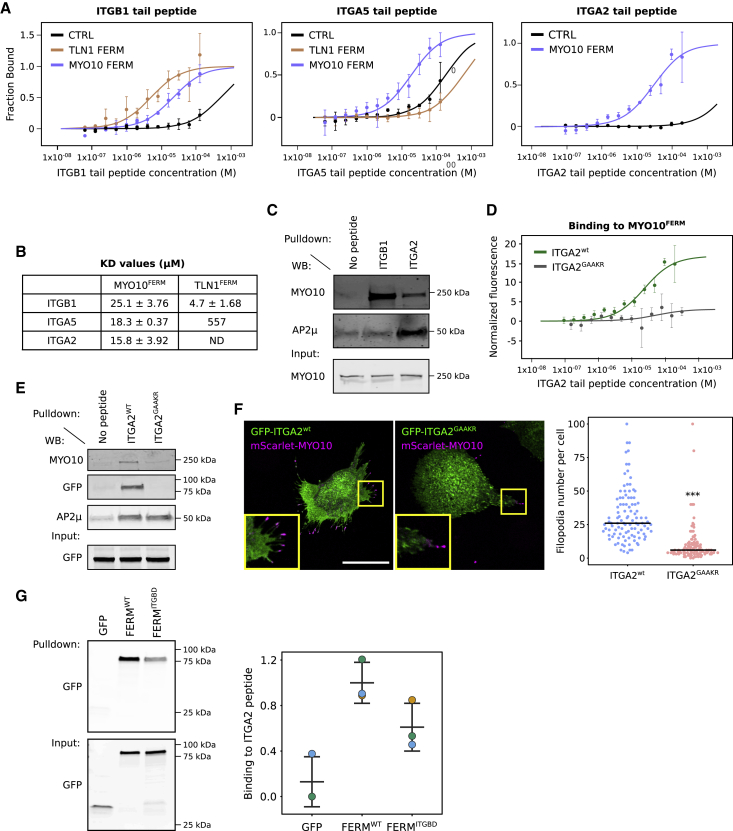
MYO10 binds to both α- and β-integrin tails (A and B) Recombinant TLN1^FERM^ and MYO10^FERM^ domain and a 6xHis CTRL peptide were labeled, and their binding to integrin tails was recorded using microscale thermophoresis. Graphs and K_D_ values were generated by pooling together three independent experiments. (C) Integrin tail pull-downs were performed from U2-OS cell lysates using magnetic beads. The recruitment of MYO10 and AP2μ was then analyzed by western blot (n = 3 biological experiments). (D) Recombinant MYO10^FERM^ was labeled, and its binding to the intracellular tails of WT ITGA2 (ITGA2^WT^) or ITGA2 mutated on the GFFKR consensus site (ITGA2^GAAKR^) was recorded using microscale thermophoresis (three independent experiments). (E) Integrin tail pull-downs were performed from cell lysate generated from U2-OS cells stably expressing EGFP-MYO10^FERM^. The recruitment of endogenous MYO10, EGFP-MYO10^FERM^, and AP2μ was then analyzed by western blot (n = 3 biological experiments). (F) CHO cells transiently expressing mScarlet-MYO10 and full-length GFP-ITGA2^WT^ or GFP-ITGA2^GAAKR^ were plated on collagen I for 2 h, fixed, and imaged using a spinning-disk microscope. Representative MIPs are displayed. Scale bar: 25 μm. The number of MYO10-positive filopodia per cell was then quantified (n > 107 cells, four biological repeats; ^∗∗∗^p < 0.001, randomization test). (G) Different EGFP-tagged MYO10 FERM domains or EGFP alone were pulled down from U2-OS lysate using α2-integrin tail peptide. MYO10 FERM recruitment to α2-integrin tail was assessed using western blot (n = 3 biological experiments).

Despite being homologous domains with high structural similarity, the functional difference between MYO10-FERM and Talin-FERM domains prompted us to compare their binding affinities to integrin cytoplasmic tails. Recombinant MYO10- and talin-FERM were expressed in bacteria, purified (Figure S6C), and their binding affinity to integrin α and β tails was measured using microscale thermophoresis (Figures 6A and 6B; see STAR Methods for details) (Jerabek-Willemsen et al., 2014). As expected, talin-FERM interacted with the β1-integrin tail (measured affinity of 4.7 μM), but not with α-integrin tails (Goult et al., 2009). This result agrees with measurements done by others using the same method (Haage et al., 2018). Interestingly, MYO10-FERM bound to the β1-integrin tail with a slightly lower affinity than talin-FERM (measured affinity of 25.1 μM) (Figures 6A and 6B). This result indicates that talin may be able to outcompete MYO10 for integrin binding.

Unexpectedly, our results indicated that, in contrast with talin-FERM, α-integrin tails also interact with MYO10-FERM *in vitro* (Figures 6A and 6B) and with endogenous MYO10 in cell lysate (Figure 6C). The ability of MYO10 to interact with both α- and β-tail peptides appeared to be specific because the clathrin adaptor AP2μ, a known α2-integrin tail-specific binder (De Franceschi et al., 2016), was pulled down only with the α2-integrin tail (Figure 6C). The MYO10-α-tail interaction was dependent on the highly conserved membrane-proximal GFFKR motif, present in most integrin α tails (De Franceschi et al., 2016). Mutation of the motif in the α2-integrin tail (FF/AA mutation, named ITGA2^GAAKR^) abolished the binding of recombinant MYO10-FERM *in vitro* (Figure 6D) and in pull-downs with full-length MYO10 (Figure 6E). Importantly, AP2μ recruitment was unaffected by the mutation (AP2μ binds to a separate motif in the α2-tail) (Figure 6E). Together, these experiments demonstrate that MYO10 binds to integrin β tails, in line with previous reports (Zhang et al., 2004; Hirano et al., 2011), revealing a previously unknown interaction between MYO10-FERM and the GFFKR motif in integrin α tails. Binding to both integrin tails has been demonstrated as a mechanism for Filamin-A-mediated integrin inactivation (Liu et al., 2015) and, thus, may be the underlying reason for the inability of MYO10-FERM alone to activate integrins.

To test the relevance of the GFFKR α-integrin tail motif in filopodia induction, we overexpressed full-length WT ITGA2 and ITGA2^GAAKR^ in CHO cells (these cells lack endogenous collagen-binding integrins) and investigated MYO10 filopodia formation on collagen I (Figure 6F). ITGA2^GAAKR^ localizes to the plasma membrane and is expressed at similar levels to WT in CHO cells (Alanko et al., 2015). ITGA2^GAAKR^-expressing cells generated fewer filopodia than cells expressing WT ITGA2, indicating that the GFFKR motif in the ITGA2 tail contributes to filopodia formation. We could not directly assess the relevance of the MYO10-α-integrin interaction to filopodia functions because the MYO10^ITGBD^ construct also displayed reduced binding toward ITGA2 (Figure 6G).

### MYO10-FERM domain fine-tunes integrin activity at filopodia tips

**Figure 7:**
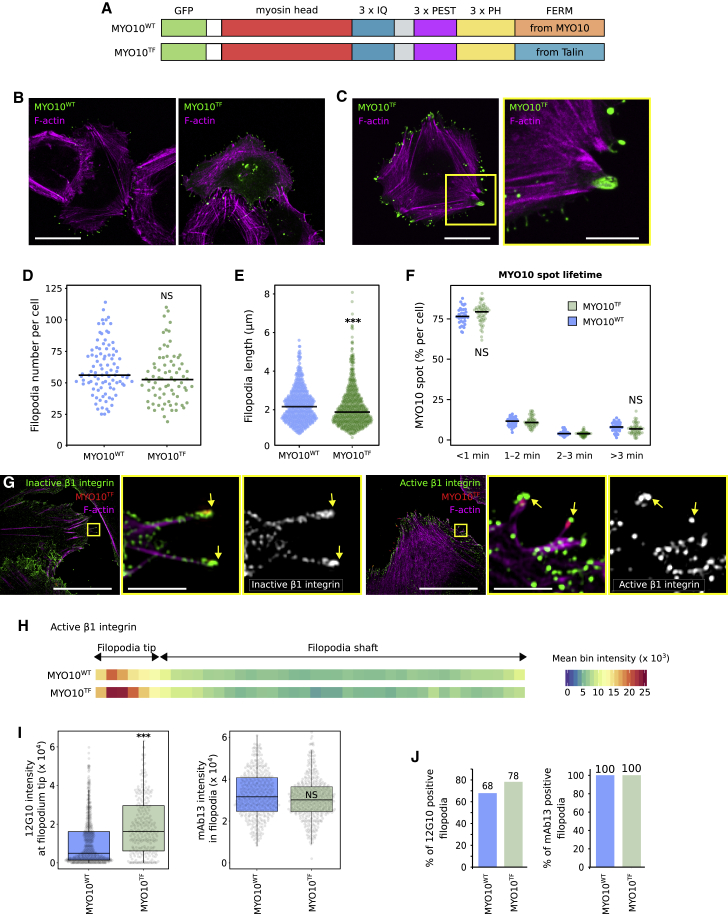
MYO10-FERM fine-tunes integrin activity at filopodia tips (A) Cartoon of the EGFP-MYO10^WT^ and EGFP-MYO10^TF^ constructs. (B–E) U2-OS cells expressing EGFP-MYO10^WT^ or EGFP-MYO10^TF^ were plated on FN for 2 h, fixed, and imaged using a spinning disk or an Airyscan microscope. (B) Representative MIPs acquired on a spinning-disk confocal are displayed; scale bar: 25 μm. (C) An image acquired on an Airyscan microscope is displayed; scale bars: (main) 25 μm; (inset) 5 μm. (D) The number of MYO10-positive filopodia per cell was quantified (n > 74 cells; three biological repeats). (E) Quantification of MYO10^WT^ and MYO10^TF^ filopodia length from SIM images (n > 512 filopodia; three biological repeats; ^∗∗∗^p < 0.001). (F) U2-OS cells expressing EGFP-MYO10^WT^ or EGFP-MYO10^TF^ were plated on FN and imaged live using an Airyscan microscope. The MYO10 spot lifetime was plotted and displayed as boxplots (three biological repeats, n > 33 cells). (G) U2-OS cells expressing EGFP-MYO10^TF^ were plated on FN for 2 h, stained for active (12G10) or inactive (mAb13) β1-integrin, and imaged using SIM. Representative MIPs are displayed; scale bars: (main) 20 μm; (inset) 2 μm. (H) Heatmap highlighting the sub-filopodial localization of active β1-integrin in cells expressing EGFP-MYO10^WT^ or EGFP-MYO10^TF^ (n > 512 filopodia; three biological repeats). (I) The average intensity of active β1-integrin (12G10) at filopodia tips and of inactive β1-integrin (mAb13) in filopodia are displayed as boxplots (^∗∗∗^p < 0.001). (J) Bar chart highlighting the percentage of MYO10^WT^ and MYO10^TF^ filopodia with detectable levels of active and inactive β1-integrin (I and J, n > 255 filopodia; three biological repeats). For all panels, p values were determined using a randomization test.

To further investigate how MYO10-FERM regulates integrin activity in filopodia and the functional differences between talin and MYO10 FERM domains, we created a chimera construct, where the FERM domain from MYO10 was replaced by the one from TLN1 (MYO10^TF^) (Figure 7A). Both MYO10^WT^ and MYO10^TF^ strongly accumulated at filopodia tips (Figures 7B and 7C). Interestingly, in a small proportion of cells (below 1%), MYO10^TF^ also localized to enlarged structures connected to stress fibers that are reminiscent of focal adhesions (Figure 7C).

Cells overexpressing MYO10^TF^ generated filopodia to the same extent as cells expressing MYO10^WT^ (Figure 7D). MYO10^TF^ filopodia were slightly shorter than MYO10^WT^ filopodia but of comparable dynamics (Figures 7E and 7F). These results show that the talin-FERM can replace the MYO10-FERM domain, and highlight an unanticipated level of interchangeability between integrin-binding FERM domains in regulating filopodia properties. Importantly, active β1-integrin accumulated more efficiently at the tips of MYO10^TF^ filopodia, and MYO10^TF^ filopodia were more likely to contain active β1-integrin at their tips than MYO10^WT^ filopodia (Figures 7G–7J). Silencing of TLN1 and TLN2 still impeded MYO10^TF^ filopodia formation, indicating that talin-FERM fused to the MYO10 motor is insufficient to substitute for the lack of endogenous full-length talin (Figures S6C and S6D). The increased amount of active β1-integrin at the tip of MYO10^TF^ filopodia is likely due to the ability of talin-FERM to activate integrin directly (Figure 5) or because talin-FERM binds to integrins with a higher affinity than MYO10-FERM (Figure 6). Altogether, our data indicate that an integrin-binding proficient FERM domain coupled to a myosin motor is required to activate, but not to transport, integrin in filopodia (Figures 2 and 5).

## Discussion

Here, we observed that active integrin accumulates at filopodia tips, while inactive integrin localizes throughout filopodia shafts. We find that integrin activation in filopodia is uncoupled from focal adhesions or the actomyosin machinery but is instead regulated by talin and MYO10. Contrary to previous assumptions, MYO10 is not required to localize integrin to filopodia, but its integrin-binding FERM domain is required for integrin activation at filopodia tips. We find, however, that, unlike talin-FERM, MYO10-FERM itself does not promote integrin activation. MYO10 and integrins also localize and modulate other cellular structures, including retraction fibers, invadopodia, growth cone filopodia, and neuronal spines (Schoumacher et al., 2010; Lin et al., 2013; Lilja and Ivaska, 2018; Peláez et al., 2019). Here, we focused on the role of MYO10 in modulating integrin in filopodia. Still, it is tempting to speculate that MYO10 may also regulate integrin activity in these other actin-rich protrusions.

We find that MYO10-FERM interaction with integrins is required to localize active integrin to filopodia tips. The simplest assumption would be that MYO10, in its typical capacity as a myosin motor, specifically transports active integrin to filopodia tips. However, our data suggest otherwise as (1) the MYO10 FERM domain alone inactivates integrins, and therefore integrins would not be in an active state during transport; (2) talin is required to localize active integrins at filopodia tips; and (3) integrin activation is thought to be a fast and tightly regulated process (Sun et al., 2019), with all evidence pointing to an on-site integrin activation mechanism in filopodia tips. In addition, direct transport of integrin by MYO10 to filopodia tips has yet to be formally observed. Our data do not exclude the possibility that MYO10 can directly transport integrin in filopodia. Testing this would require performing two-color, single-molecule imaging of MYO10 and integrin to see if they move toward filopodia tips together. However, we find integrins abundantly in filopodia regardless of the MYO10 status. Altogether, we propose that inactive integrins localize along the filopodia plasma membrane via membrane diffusion and are activated at filopodia tips in a two-step process by MYO10 and talin. In this model, MYO10 could tether integrins at filopodia tips because of its motor domain and provide resistance against the actin retrograde flow present in filopodia (Bornschlögl et al., 2013; Lidke et al., 2005) allowing sufficient time for talin-mediated activation.

The precise mechanisms favoring integrin binding to MYO10 or talin in filopodia remain to be elucidated. One possibility is that talin-FERM outcompetes MYO10-FERM. Indeed, our *in vitro* experiments indicate that talin-FERM has, in solution, a higher affinity for integrin β tail compared with MYO10-FERM. In addition, talin affinity for β-integrin tails will be even stronger in cells because of the presence of negatively charged membrane phosphoinositides that interact with talin-FERM (Chinthalapudi et al., 2018; De Franceschi et al., 2018), and which are known to accumulate at filopodia tips (Jacquemet et al., 2019). Interestingly, although MYO10 and talin FERM domains structurally adopt a very similar fold, we find that these two FERM domains are functionally distinct. MYO10-FERM is not capable of directly activating integrin and can interact with both integrin tails. Yet, remarkably, swapping MYO10-FERM with talin-FERM fully supported filopodia function and integrin activation at filopodia tips, suggesting unanticipated interchangeability between these FERM domains in spatially regulating integrin activation in filopodia. Other FERM domain-containing myosins, including MYO7 and MYO15, also localize to filopodia tips (Jacquemet et al., 2019; Arthur et al., 2019), where their roles are mostly unknown; future work will examine the contribution of these unconventional myosins to filopodia functions.

## STAR★Methods

### Key resources table

**Table:** 

REAGENT or RESOURCE	SOURCE	IDENTIFIER
**Antibodies**		

Mouse anti-human active b1 integrin (12G10)	In house	RRID:AB_775726
Mouse anti-human β1 integrin (HUTS21)	BD Biosciences	catalog number: 556048; RRID:AB_396319
Rat anti-human β1 integrin (9EG7)	BD Biosciences	catalog number: 553715; RRID:AB_395001
Mouse anti-human β1 integrin (4B4)	Beckman Coulter	catalog number: 6603113; RRID:AB_10638675
Rat anti-human β1 integrin (mAb13)	In house	RRID:AB_394479
Mouse anti-human β1 integrin (P5D2)	Developmental studies hybridoma bank	catalog number: p5d2; RRID:AB_528308
Mouse monoclonal anti-hamster ɑ5 integrin (PB1)	Developmental studies hybridoma bank	catalog number: pb1; RRID:AB_528300
Mouse monoclonal anti-human TLN1 (97H6)	Novus Biologicals	catalog number: NBP2-50320; RRID:AB_11159092
Mouse monoclonal anti-human TLN2 (68E7)	Novus Biologicals	catalog number: NBP2-50322
Mouse monoclonal anti-β-actin (AC-15)	Merck	catalog number: A1978; RRID:AB_476692
Mouse monoclonal anti-PAX (349)	BD Biosciences	catalog number: 610051; RRID:AB_397463
Rabbit monoclonal anti-AP2μ	Novus Biological	catalog number: EP2695Y; RRID:AB_2258308
Rabbit polyclonal anti-GFP	Abcam	catalog number: Ab290; RRID:AB_303395
Rabbit polyclonal anti-MYO10	Novus Biologicals	catalog number: 22430002; RRID:AB_2148055
Rabbit polyclonal anti-kindlin-1 (recognizes kindlin 1 and 2)	Abcam	catalog number: ab68041; RRID:AB_1603823

**Chemicals, peptides, and recombinant proteins**		

RO-3306	Merck	catalog number: SML0569
Blebbistatin	Stemcell technologies	catalog number: 72402
Bovine plasma fibronectin	Merck	catalog number: 341631
Collagen I	Merck	catalog number: C8919-20ML
Wild-type β1-integrin tail (KLLMIIHDRRE FAKFEKEKMNAKWDTGENPIYKSAVTT VVNPKYEGK) custom peptide	*LifeTein*	N/A
β1-integrin tail where the NPXY motif is deleted (KLLMIIHDRREFAKFEKEKMNA KWDTGEN) custom peptide	*LifeTein*	N/A
conserved region of the α2-integrin tail (WKLGFFKRKYEKM) custom peptide	*LifeTein*	N/A
α2-integrin tail peptide where the GFFKR motif is mutated (WKLGAAKRKYEKM) custom peptide	*LifeTein*	N/A
α5-integrin tail (KLGFFKRSLPYGTAM EKAQLKPPATSDA) custom peptide	*LifeTein*	N/A

**Experimental models: Cell lines**		

U2-OS osteosarcoma cells	Leibniz Institute DSMZ-German Collection of Microorganisms and Cell Cultures, Braunschweig DE	catalog number: ACC 785
MDA-MB-231 triple-negative human breast adenocarcinoma	ATCC	catalog number: HTB-26
CHO-K1 cells	ATCC	catalog number: CCL-61

**Oligonucleotides**		

siACTN1 #5	QIAGEN	catalog number: Hs_ACTN1_5, SI00299131
siACTN1 #2	QIAGEN	catalog number: Hs_ACTN1_2, SI00021917
siTNS3 #1	QIAGEN	catalog number: Hs_TENS1_1, SI00134372
siTNS3 #2	QIAGEN	catalog number: Hs_TNS3_2, SI02778643
siTNS1 #3	QIAGEN	catalog number: Hs_TNS_3, SI00134106
siTNS1 #4	QIAGEN	catalog number: Hs_TNS_4, SI00134113
siFERMT1 #5	QIAGEN	catalog number: Hs_C20orf42_5, SI04269181
siFERMT1 #7	QIAGEN	catalog number: Hs_C20orf42_7, SI04307219
siFERMT1 #8	QIAGEN	catalog number: Hs_C20orf42_8, SI04352978
siFERMT2 #1	*QIAGEN*	catalog number: Hs_FERMT2_1, SI04952542
siFERMT2 #3	*QIAGEN*	catalog number: Hs_FERMT2_3, SI04952556
siCIB1 #5	*QIAGEN*	catalog number: Hs_CIB1_5, SI02657102
siCIB #7	*QIAGEN*	catalog number: Hs_CIB1_7, SI03164476
siSHARPIN #2	*QIAGEN*	catalog number: Hs_SHARPIN_2, SI00140182
siSHARPIN #5	*QIAGEN*	catalog number: Hs_SHARPIN_5, SI03067344
siITGB1BP1 #5	QIAGEN	catalog number: Hs_ITGB1BP1_5, SI03129385
siITGB1BP1 #8	QIAGEN	catalog number: Hs_ITGB1BP1_8, SI04332832
*siTLN1 #2*	*QIAGEN*	*catalog number: Hs_TLN1_2, SI00086968*
*siTLN1 #3*	*QIAGEN*	*catalog number: Hs_TLN1_3, SI00086975*
siTLN2 #3	QIAGEN	catalog number: Hs_TLN2_3, SI00109277
siTLN2 #4	Dharmacon	catalog number: LQ’012909-00-0002
siMYO10 #5	QIAGEN	catalog number: Hs_MYO10_5, SI04158245
siMYO10 #6	QIAGEN	catalog number: Hs_MYO10_6, SI04252822
siMYO10 #7	QIAGEN	catalog number: Hs_MYO10_7, SI05085507
Primers for TNS1 (cca gac acc cac ctg act tag; ttg gtg cat tct cag tgg tg; probe 58)	IDT	N/A
Primers for ACTN1 (gcc tca tca gct tgg gtt at; cat gat gcg ggc aaa ttc; probe 7)	IDT	N/A
Primers for FERMT1 (aga cgt cac act gag agt atc tgg; tct gac cag tct tgg gat ata ttg; probe 25)	IDT	N/A
Primers for TNS3 (agg ctg cct gac aca gga; agg ggc tgt tca gca gag; probe 57)	IDT	N/A
Primers for TLN1 (ccc tta cct ggg gag aca at; gag ctc acg gct ttg gtg; probe 61)	IDT	N/A
Primers for CIB1 (agt tcc agc acg tca tct cc; gct gct gtc aca gga caa tc; probe 17)	IDT	N/A
Primers for ITGB1BP (ttg aag ggc cat tag acc tg; gaa caa aag gca act ttc cat c; probe 61)	IDT	N/A
Primers for FERMT2 (taa aa cat ggc gtt tca gca; cat ctg caa act cta cgg tgac; probe 48)	IDT	N/A
Primers for SHARPIN (ccc tgg ctg tga gat gtg ta; ggc cac tct ccc ctt gta ac; probe 83)	IDT	N/A
Primers for FLNA (gtc acc ggt cgc tct cag; agg gga cgg ccc ttt aat; probe 32)	IDT	N/A
Primers for TLN2 (ggt cat ggt tgg gca gat; gca tgc ttg tgt tga tgg tc; probe 40)	IDT	N/A

**Recombinant DNA**		

EGFP-MYO10^FERM^	This study, Addgene	catalog number: 145140
His-tagged MYO10^FERM^	This study	N/A
EGFP-MYO10-FERM^ITGBD^	This study	N/A
EGFP-MYO10^ΔF^	This study, Addgene	catalog number: 145816
mScarlet-I-MYO10^ΔF^	This study, Addgene	catalog number: 145139
EGFP-MYO10^TF^	This study, Addgene	catalog number: 145141
EGFP-MYO10^ITGBD^	This study	N/A
EGFP-MYO10^ΔF2F3^	This study	N/A
EGFP-MYO10^ΔF3^	This study	N/A
EGFP-MYO10	Addgene (Emanuel Strehler) (Bennett et al., 2007)	catalog number: 47608
CRK-GFP	Addgene (Ken Yamada)	catalog number: 50730
VASP-GFP	Addgene (Michael Davidson)	catalog number: 54297
DIAPH3-GFP	Addgene (Michael Davidson)	catalog number: 54158
BCAR1-GFP	Daniel Rösel (Charles University in Prague, Czech Republic) (Braniš et al., 2017).	N/A
FERMT2-GFP	Maddy Parsons (King’s College London, UK)	N/A
GFP-ITGA2	(Pellinen et al., 2006)	N/A
GFP-ITGA2^GAAKR^	(Pellinen et al., 2006)	N/A
mScarlet-MYO10	(Jacquemet et al., 2019)	N/A
GFP-TLN1	(Kopp et al., 2010)	N/A
GFP-TLN1^FERM^	(Goult et al., 2010)	N/A
His-TLN1^FERM^	(Goult et al., 2010)	N/A

**Software and algorithms**		

FiloMAP	(Jacquemet et al., 2019)	https://github.com/guijacquemet/FiloMAP
RStudio (1.3.1093)	Foundation for Open Access Statistics.	https://www.rstudio.com/
Fiji (2.1)	(Schindelin et al., 2012)	https://fiji.sc/
TrackMate	(Tinevez et al., 2017)	https://imagej.net/plugins/trackmate/
PlotsOfDifferences	(Goedhart, 2019)	https://huygens.science.uva.nl/PlotsOfDifferences/
PlotsOfData	(Postma and Goedhart, 2019)	https://huygens.science.uva.nl/PlotsOfData/
SuperPlotsofData	(Goedhart, 2021)	https://huygens.science.uva.nl/SuperPlotsOfData/
MO.Affinity software	NanoTemper	https://nanotempertech.com/monolith-mo-control-software/
SlideBook 6	*Intelligent Imaging Innovations, Inc*	https://www.intelligent-imaging.com/slidebook
SoftWorx	*GE Healthcare*	*N/A*
Zen Black (2.3)	*Zeiss*	https://www.zeiss.com/microscopy/int/products/microscope-software/zen.html

**Other**		

VECTASHIELD	Vector laboratories	catalog number: H-1000

### Resource availability

#### Lead contact

Further information and requests for resources and reagents should be directed to and will be fulfilled by the lead contact, Guillaume Jacquemet (guillaume.jacquemet@abo.fi).

#### Materials availability

Several of the plasmids generated in this study have been deposited to Addgene: EGFP-MYO10^FERM^ (catalog number: 145140), EGFP-MYO10^ΔF^ (catalog number: 145816), mScarlet-I-MYO10^ΔF^ (catalog number: 145139), EGFP-MYO10^TF^ (catalog number: 145141). The other plasmids generated in this study will also be available on Addgene soon.

### Experimental model and subject details

U2-OS (human osteosarcoma) and MDA-MB-231 (triple-negative human breast adenocarcinoma) cells were grown in DMEM (Dulbecco’s Modified Eagle’s Medium with HEPES modification; Sigma, D1152) supplemented with 10% fetal bovine serum (FCS) (Biowest, S1860). CHO cells were cultured in alpha-MEM, supplemented with 5% FCS and L-glutamine.

### Method details

#### Plasmids and transfection

U2-OS, MDA-MB-231, and CHO cells were transfected using Lipofectamine 3000 and the P3000TM Enhancer Reagent (Thermo Fisher Scientific) according to the manufacturer’s instructions. The U2-OS MYO10-GFP lines were generated by transfecting U2-OS cells using Lipofectamine 3000 (ThermoFisher Scientific), selected using Geneticin (ThermoFisher Scientific; 400 μg.ml^-1^ final concentration) and sorted for green fluorescence using a fluorescence-assisted cell sorter (FACS). All cell lines tested negative for mycoplasma.

#### Plasmids

The construct encoding the EGFP-tagged MYO10-FERM domain (EGFP-MYO10^FERM^) was designed using the boundaries from the MYO10-FERM crystal structure (Wei et al., 2011). The MYO10 coding region 1480-2053 was amplified by PCR (primers: 5′-ATT AGA GAA TTC AAC CCG GTG GTC CAG TGC-3′, 5′-ATT AGA GGT ACC TCA CCT GGA GCT GCC CTG-3′), and the resulting PCR products were ligated into pEGFP-C1 using the EcoRI and KpnI restriction sites. To generate the EGFP-MYO10-FERM^ITGBD^ mutant, a synthetic DNA sequence (gene block, IDT) encoding the MYO10 FERM domain (as indicated above) containing the appropriate mutations (S2001_F2002insA/T2009D) was inserted into pEGFP-C1 using the EcoRI/KpnI restriction sites. To generate the His-tagged MYO10^FERM^ plasmid, the MYO10-FERM domain (boundaries 1504-2058 in MYO10) was amplified by PCR (primers: 5′-ATT AGA GCG GCC GCA CCG ATC GAC ACC CCC AC, 5′-ATT AG AGA ATT CTC ACC TGG AGC TGC CCT G) and introduced in pET151 using the NotI and EcoRI restriction sites.

The MYO10 MyTH/FERM deletion construct (EGFP-MYO10^ΔF^) was generated by introducing a premature stop codon in full-length EGFP-MYO10 (boundaries 1-1512 in MYO10) using a gene block (IDT). The gene block was inserted in EGFP-MYO10 using the PvuI and XbaI restriction sites.

The mScarlet-I-MYO10^ΔF^ construct was created from EGFP-MYO10^ΔF^ by swapping the fluorescent tag. The mScarlet-I (Bindels et al., 2017) coding sequence, acquired as a gene block (IDT), was inserted in EGFP-MYO10^ΔF^ using the NheI and KpnI restriction sites.

The MYO10/TLN1 chimera construct (EGFP-MYO10^TF^) was generated by swapping the MYO10-FERM domain (boundaries 1504-2056 in MYO10) with the TLN1-FERM domain (boundaries 1-398 in TLN1) using a gene block (IDT). The gene block was inserted in EGFP-MYO10 using the PvuI and XbaI restriction sites.

The EGFP-MYO10^ITGBD^ construct was generated by replacing the wild-type MYO10-FERM domain (boundaries 1504-2056 in MYO10) with a MYO10 FERM domain containing the required mutations (S2001_F2002insA/T2009D) using a gene block (IDT). The gene block was inserted in EGFP-MYO10 using the PvuI and XbaI restriction sites.

The EGFP-MYO10^ΔF2F3^ and EGFP-MYO10^ΔF3^ constructs were generated by replacing the wild-type MYO10-FERM domain (boundaries 1504-2056 in MYO10) with truncated MYO10 FERM domains where the F2-F3 or F3 FERM lobes are deleted using gene blocks (IDT). The gene blocks were inserted in EGFP-MYO10 using the PvuI and XbaI restriction sites. The final boundaries compared to full-length MYO10 are 1-1794 for MYO10^ΔF2F3^ and 1-1951 for MYO10^ΔF3^.

#### siRNA-mediated gene silencing

The expression of proteins of interest was suppressed using 83 nM siRNA and lipofectamine 3000 (Thermo Fisher Scientific) according to the manufacturer’s instructions. All siRNAs used were purchased from QIAGEN. siMYO10 #7 targets the 3′ UTR of the MYO10 mRNA and therefore does not affect the expression of MYO10 constructs.

#### SDS–PAGE and quantitative western blotting

Purified proteins or protein extracts were separated under denaturing conditions by SDS–PAGE and transferred to nitrocellulose membranes using Trans-Blot Turbo nitrocellulose transfer pack (Bio-Rad, 1704159). Membranes were blocked for 45 min at room temperature using 1x StartingBlock buffer (Thermo Fisher Scientific, 37578). After blocking, membranes were incubated overnight with the appropriate primary antibody (1:1000 in PBS), washed three times in TBST, and probed for 40 min using a fluorophore-conjugated secondary antibody diluted 1:5000 in the blocking buffer. Membranes were washed three times using TBST, over 15 min, and scanned using an Odyssey infrared imaging system (LI-COR Biosciences).

#### siRNA screen

96-well glass-bottom plates (Cellvis, P96-1.5H-N) were first coated with a solution of poly-D-lysine (10 μg/ml in PBS, Sigma-Aldrich, A-003-M) at 4°C overnight. Plates were then washed with PBS and coated with a solution containing 10 μg/ml of bovine fibronectin (in PBS) also at 4°C overnight. Excess fibronectin was washed away with PBS.

U2-OS cells stably expressing MYO10-GFP were silenced for the gene of interest using a panel of siRNAs (QIAGEN flexiplate, 1704159) using Lipofectamine 3000 (Thermo Fisher Scientific, L3000075). 48 h post silencing, cells were trypsinized and plated on both fibronectin-coated 96-well glass-bottom plates and 96-well plastic-bottom plates in full culture medium. Cells plated in the plastic-bottom plates were allowed to spread for two hours before being lysed using an RNA extraction buffer. RNAs were then purified and the silencing efficiency of each siRNA was validated by qPCR analysis.

Cells plated in the glass-bottom plates were allowed to spread for two hours and fixed with a warm solution of 4% paraformaldehyde (PFA; Thermo Scientific, 28906). After washing, the samples were incubated with a solution of 1 M glycine (30 min, in PBS) and then for one hour in a solution containing phalloidin–Atto647N (1/400 in PBS, Thermo Fisher Scientific, 65906) and DAPI (0.5 μg/ml in PBS, Thermo Fisher Scientific, D1306). The 96-well glass-bottom plates were then imaged using a spinning-disk confocal microscope equipped with a 40x objective. Images were analyzed using Fiji (Schindelin et al., 2012). Briefly, images were opened and, after background subtraction and normalization, MYO10 spots were automatically detected using Michael Schmid’s ‘Find maxima’ plugin. As inactive MYO10 is known to accumulate in rab7 vesicles (Plantard et al., 2010), to obtain an accurate number of filopodia-specific MYO10 spots, intracellular MYO10 spots were excluded from the analysis. Intracellular MYO10 spots were automatically filtered by masking the cells using the F-actin staining. The remaining spots per field of view were counted.

#### RNA extraction, cDNA preparation, and Taq-Man qPCR

Total RNA extracted using the NucleoSpin RNA Kit (Macherey-Nagel, 740955.240C) was reverse transcribed into cDNA using the high-capacity cDNA reverse transcription kit (Applied Biosystems, Thermo Fisher Scientific, 43-688-14) according to the manufacturer’s instructions. The TaqMan primer sequences and associated universal probes were generated using ProbeFinder (version 2.53, Roche). The primers themselves were ordered from IDT, and the TaqMan fast advanced master mix (Thermo Fisher Scientific, 4444557) was used to perform the qPCR reactions according to the manufacturer’s instructions. qPCR reactions were analyzed with the 7900HT fast RT-PCR System (Applied Biosystems), and the results were analyzed using the RQ Manager Software (Applied Biosystems). Relative expression was calculated by the 2^-ΔΔCT^ method. GAPDH mRNA levels were used to normalize data between experiments and conditions.

#### Generation of filopodia maps

U2-OS cells transiently expressing the constructs of interests were plated on high tolerance glass-bottom dishes (MatTek Corporation, coverslip #1.7) pre-coated first with Poly-L-lysine (10 μg/ml, 1 h at 37°C) and then with bovine plasma fibronectin (10 μg/ml, 2 h at 37°C). After 2 h, samples were fixed and permeabilized simultaneously using a solution of 4% (wt/vol) PFA and 0.25% (vol/vol) Triton X-100 for 10 min. Cells were then washed with PBS, quenched using a solution of 1 M glycine for 30 min, and, when appropriate, incubated with the primary antibody for 1 h (1:100). After three washes, cells were incubated with a secondary antibody for 1 h (1:100). Samples were then washed three times and incubated with SiR-actin (100 nM in PBS; Cytoskeleton; catalog number: CY-SC001) at 4°C until imaging (minimum length of staining, overnight at 4°C; maximum length, one week). Just before imaging, samples were washed three times in PBS and mounted in Vectashield (Vector Laboratories).

To map the localization of each protein within filopodia, images were first processed in Fiji (Schindelin et al., 2012) and data analyzed using R as previously described (Jacquemet et al., 2019). Briefly, in Fiji, the brightness and contrast of each image was automatically adjusted using, as an upper maximum, the brightest cellular structure labeled in the field of view. In Fiji, line intensity profiles (1-pixel width) were manually drawn from filopodium tip to base (defined by the intersection of the filopodium and the lamellipodium). To avoid any bias in the analysis, the intensity profile lines were drawn from a merged image. All visible filopodia in each image were analyzed and exported for further analysis (export was performed using the ‘‘Multi Plot’’ function). For each staining, line intensity profiles were then compiled and analyzed in R. To homogenize filopodia length; each line intensity profile was binned into 40 bins (using the median value of pixels in each bin and the R function ‘‘tapply’’). Using the line intensity profiles, the percentage of filopodia positive for active β1 at their tip was quantified. A positive identification was defined as requiring at least an average value of 5000 (values between 0-65535) within the bins defining the filopodium tip (identified using MYO10 staining). The map of each protein of interest was created by averaging hundreds of binned intensity profiles. The length of each filopodium analyzed was directly extracted from the line intensity profiles.

The preferential recruitment of active and inactive β1 integrin to filopodia tips or shafts was assessed by calculating an enrichment ratio where the averaged intensity of the β1 integrin species at the filopodium tip (bin 1-6) was divided by the averaged intensity at the filopodium shaft (bin 7-40). This enrichment ratio was calculated for each filopodium analyzed and the results were displayed as Tukey boxplots.

#### Quantification of filopodia numbers and dynamics

For the filopodia formation assays, cells were plated on fibronectin-coated glass-bottom dishes (MatTek Corporation) for 2 h. Samples were fixed for 10 min using a solution of 4% PFA, then permeabilized using a solution of 0.25% (vol/vol) Triton X-100 for 3 min. Cells were then washed with PBS and quenched using a solution of 1 M glycine for 30 min. Samples were then washed three times in PBS and stored in PBS containing SiR-actin (100 nM; Cytoskeleton; catalog number: CY-SC001) at 4°C until imaging. Just before imaging, samples were washed three times in PBS. Images were acquired using a spinning-disk confocal microscope (100x objective). The number of filopodia per cell was manually scored using Fiji (Schindelin et al., 2012).

To study filopodia stability, U2-OS cells expressing MYO10-GFP were plated for at least 2 h on fibronectin before the start of live imaging (pictures taken every 5 s at 37°C, on an Airyscan microscope, using a 40x objective). All live-cell imaging experiments were performed in normal growth media, supplemented with 50 mM HEPES, at 37°C and in the presence of 5% CO_2_. Filopodia lifetimes were then measured by identifying and tracking all MYO10 spots using the Fiji plugin TrackMate (Tinevez et al., 2017). In TrackMate, the LoG detector (estimated bob diameter = 0.8 mm; threshold = 20; subpixel localization enabled) and the simple LAP tracker (linking max distance = 1 mm; gap-closing max distance = 1 mm; gap-closing max frame gap = 0) were used.

#### Light microscopy setup

The spinning-disk confocal microscope (spinning-disk confocal) used was a Marianas spinning-disk imaging system with a Yokogawa CSU-W1 scanning unit on an inverted Zeiss Axio Observer Z1 microscope controlled by SlideBook 6 (Intelligent Imaging Innovations, Inc.). Images were acquired using either an Orca Flash 4 sCMOS camera (chip size 2,048 × 2,048; Hamamatsu Photonics) or an Evolve 512 EMCCD camera (chip size 512 × 512; Photometrics). Objectives used were a 40x water (NA 1.1, LD C-Apochromat, Zeiss), a 63 × oil (NA 1.4, Plan-Apochromat, M27 with DIC III Prism, Zeiss) and a 100x oil (NA 1.4 oil, Plan-Apochromat, M27) objective.

The structured illumination microscope (SIM) used was DeltaVision OMX v4 (GE Healthcare Life Sciences) fitted with a 60x Plan-Apochromat objective lens, 1.42 NA (immersion oil RI of 1.516) used in SIM illumination mode (five phases x three rotations). Emitted light was collected on a front-illuminated pco.edge sCMOS (pixel size 6.5 mm, readout speed 95 MHz; PCO AG) controlled by SoftWorx.

The confocal microscope used was a laser scanning confocal microscope LSM880 (Zeiss) equipped with an Airyscan detector (Carl Zeiss) and a 40x oil (NA 1.4) objective. The microscope was controlled using Zen Black (2.3), and the Airyscan was used in standard super-resolution mode.

#### Integrin activity assays

CHO cells detached using Hyclone HyQTase (Thermo Fisher Scientific, SV300.30.01), washed with Tyrode’s Buffer (10 mM HEPES-NaOH, pH 7.5, 137 mM NaCl, 2.68 mM KCl, 0.42 mM NaH_2_PO_4_, 1.7 mM MgCl_2_, 11.9 mM NaHCO_3_, 5 mM glucose, and 0.1% BSA) and pretreated for 10 min with or without 5 mM EDTA in serum-free alpha-MEM media. Cells were then incubated for 40 min with Alexa Fluor 647 labeled fibronectin fragment (FN 7-10). After washing away the unbound fibronectin using Tyrode’s buffer, cells were fixed with 4% PFA (in PBS) for 10 min at room temperature. Part of the HyQTase treated cells were also fixed with 4% PFA (in PBS) and stained with an anti-hamster anti-ɑ5 integrin antibody to detect total ITGA5 levels in cells (2 h at 4C, 1:10 in PBS, antibody PB1, Developmental studies hybridoma bank) and with an Alexa Fluor 647-conjugated secondary antibody (45 min at RT, 1:200 in PBS, Thermo Fisher Scientific, A-21235). Fluorescence intensity was recorded using FACS (BD LSRFortessa). Data were gated and analyzed using the Flowing Software (https://bioscience.fi/services/cell-imaging/flowing-software/). The integrin activity index (IA) was calculated for each condition as a ratio AI = (F−F_EDTA_)/(F_PB1_), where F = FN7-10 signal, F_EDTA_ = FN7-10 signal in EDTA treated cells and F_PB1_ = ɑ5 integrin signal.

MDA-MB-231 and U2-OS cells detached using Hyclone HyQTase (Thermo Fisher Scientific, SV300.30.01) were fixed with 4% PFA (in PBS) for 10 min and stained for active (antibody 9EG7) and total β1 integrin (antibody P5D2) overnight at 4°C. Cells were then stained with the appropriate Alexa Fluor 647-conjugated secondary antibody (45 min at RT, 1:200, Thermo Fisher Scientific) and the fluorescence was recorded using FACS. Data were gated and analyzed using the Flowing Software (https://bioscience.fi/services/cell-imaging/flowing-software/) and the integrin activity (IA) was calculated as indicated below where F_9EG7_ and F_P5D2_ are the signals intensities of the 9EG7 and P5D2 stainings, respectively. F_2nd Ab_ corresponds to the signal intensity recorded when the cells are stained with only the secondary antibody.

$$\text{IA}\;=\;\left({\text{F}}_{9\text{EG}7}-\;{\text{F}}_{2\text{nd Ab}}\right)\;/\;\left({\text{F}}_{\text{P}5\text{D}2}-\;{\text{F}}_{2\text{nd Ab}}\right)$$

#### Cell spreading assay

The xCELLigence RTCA instrument (Roche) was used to measure cell adhesion on fibronectin in real-time (Hamidi et al., 2017). The RTCA instrument uses gold-bottom electrode plates to measure the impedance between two electrodes. This is expressed as an arbitrary cell index value. The xCELLigence 96-well plates (Acea Biosciences, E-Plate VIEW 96 PET, 00300600900) were coated with a solution of 20 μg/ml of poly-D-lysine (in PBS) for 1 h at 37°C, washed with PBS, and coated with a solution of 10 μg/ml fibronectin (in PBS) for 1 h at 37°C. Plates were then blocked using a solution of 1% BSA (in PBS) for 1 h in RT. After 2 PBS washes, 15000 cells were seeded into each well in a serum-free culture medium. The cell index was recorded over time.

#### Recombinant protein expression and purification

The *E. coli* BL-21(DE3) strain was transformed with IPTG inducible, His-tagged expression constructs, and the transformed bacteria were grown at 37°C in LB media supplemented with ampicillin (1 mg/ml) until OD600 was 0.6-0.8. Protein expression was then induced using IPTG (0.5 mM), and the temperature was lowered to 25°C. Cells were harvested after 5 h by centrifugation (20 min at 6000 g). Bacteria were then resuspended in a resuspension buffer (1x TBS, cOmplete protease inhibitor tablet (Roche, cat. no. 5056489001), 1x AEBSF inhibitor, 1x PMSF, RNase 0.05 mg/ml, DNase 0.05 mg/ml). To lyse the bacteria, a small spoonful of lysozyme and 1x BugBuster (Merck Millipore, cat. no. 70584-4) were added, and the suspension was agitated for 30 min at 4°C. Cell debris was pelleted using a JA25.5 rotor at 20000 rpm for 1 h. His-tagged proteins were batch purified from the supernatant using a Protino Ni-TED 2000 column (Macherey Nagel, cat. no. 745120.25) according to the manufacturer’s instructions. Proteins were eluted using the elution buffer provided with the kit supplemented with 1 mM AEBSF. For each purified protein, several 1 mL fractions were collected, ran on a 4%–20% protein gel (Bio-Rad Mini-PROTEAN TGX, #4561093), stained with InstantBlue® (Expedeon, ISB1L), and the fractions abundant in tagged protein were combined. Imidazole was removed in a buffer exchange overnight at 4°C and 1 mM AEBSF was added to the imidazole-free protein. Proteins were stored at 4°C for up to one week.

#### Whole-mount immuno-SEM

U2-OS cells expressing MYO10-GFP were plated for 2 h on fibronectin-coated coverslips and fixed with a solution of 4% PFA (in 0.1 M HEPES, pH 7.3) for 30 min. After washing and quenching with 50 mM NH_4_Cl (in 0.1 M HEPES), non-specific binding was blocked with a buffer containing 2% BSA (in 0.1 M HEPES). Samples were then labeled using the appropriate primary antibody (1:10 in 0.1 M HEPES) for 30 min, washed, and labeled with a gold conjugated secondary antibody (1:50 in 0.1 M HEPES, 30 nm gold particles, BBI solutions, EM.GAF30) for 30 min. After immunolabeling, the samples were washed, and post-fixed with a solution of 2.5% glutaraldehyde and 1% buffered osmium tetroxide prior to dehydration and drying using hexamethyldisilazane. The dried samples were mounted on SEM stubs and sputter-coated with carbon. The micrographs were acquired with FEI Quanta FEG 250 microscope with SE and vC detectors (FEI Comp.) using an acceleration voltage of 5.00 kV and a working distance ranging from 7.7 to 10.9 mm.

To compare the distribution of active and inactive integrin from EM images, we manually measured the distance between each detected gold particle and the filopodium tip using Fiji. Results were then plotted as a probability density function where the area under the curve represents 100% probability. A bootstrap version of the univariate Kolmogorov-Smirnov test was then used to assess statistical significance (using Rstudio). Importantly, filopodia length was not normalized in these analyses.

#### Integrin tail pull-downs

For each pulldown, 20 μL of streptavidin Dynabeads (MyOne Streptavidin C1, Invitrogen, 65001) were incubated, for 30 min, on ice, with the appropriate biotinylated integrin tail peptides (50 ug per sample) (LifeTein). U2-OS cells were washed twice with cold PBS and lysed on ice with a buffer containing 40 mM HEPES, 75 mM NaCl, 2 mM EDTA, 1% NP-40, a cOmplete protease inhibitor tablet (Roche, 5056489001) and a phosphatase-inhibitor tablet (Roche, 04906837001). Samples were cleared by centrifugation (13,000 g, 10 min) and incubated with the streptavidin Dynabeads for 2 h at 4°C. Beads were washed three times with a washing buffer (50 mM Tris-HCl pH 7.5, 150 mM NaCl, 1% (v/v) NP-40), and proteins bound to the beads were eluted using SDS sample buffer and heated for 5-10 min at 90°C. Results were analyzed using western blots. Integrin peptides used were wild-type β1-integrin tail (KLLMIIHDRREFAKFEKEKMNAKWDTGENPIYKSAVTTVVNPKYEGK), the β1-integrin tail where the NPXY motif is deleted (KLLMIIHDRREFAKFEKEKMNAKWDTGEN), the conserved region of the α2-integrin tail (WKLGFFKRKYEKM), the conserved region of α2-integrin tail peptide where the GFFKR motif is mutated (GAAKR mutant, WKLGAAKRKYEKM) and the wild-type α5-integrin tail (KLGFFKRSLPYGTAMEKAQLKPPATSDA).

#### Microscale thermophoresis

Recombinant His-tagged proteins were labeled using the Monolith His-Tag Labeling Kit RED-tris-NTA (NanoTemper, MO-L008). In all experiments, the labeled His-tagged recombinant proteins were used at a concentration of 20 nM while the integrin tail peptides were used at increasing concentration. Kd values were calculated using the equation provided below (Equation 1), where Kd is the dissociation constant, [A] the concentration of the free fluorescent molecule, [L] the concentration of the free ligand, [AL] the concentration of the AL-complex. [A0] is the known concentration of the fluorescent molecule and [L0] is the known concentration of added ligand. This leads to a quadratic fitting function for [AL]:

Eq.1 [AL]=1/2∗(([A0]+[L0]+Kd)−(([A0]+[L0]+Kd)2−4∗[A0]∗[L0])1/2)

Alternatively, binding was also expressed as a change in MST signal (normalized fluorescence ΔFnorm). This is defined as a ratio:

Eq.2 ΔFnorm = F1/F0

Where F0 is the fluorescence prior and F1 after IR laser activation.

All binding data were analyzed using MO.Control and MO.Affinity software (NanoTemper).

### Quantification and statistical analysis

Randomization tests were performed using the online tool PlotsOfDifferences (https://huygens.science.uva.nl/PlotsOfDifferences/) (Goedhart, 2019). Dot plots were generated using PlotsOfData (Postma and Goedhart, 2019). SuperPlots were generated using SuperPlotsofData (Lord et al., 2020; Goedhart, 2021). Bar plots with visualized data points, time-series data, and density plots were generated using R (https://www.r-project.org/), Rstudio (Integrated Development for R. RStudio, Inc., Boston, MA. https://www.rstudio.com/) and ggplot2 (Wickham, 2016). The univariate Kolmogorov-Smirnov test was performed using Rstudio. Other statistical analyses were performed using Google sheets except for the one-sample t test which was performed using an online calculator (https://www.socscistatistics.com/tests/tsinglesample/default.aspx).

## Data Availability

The authors declare that the data supporting the findings of this study are available within the article and from the authors upon request. Any additional information required to reanalyze the data reported in this paper is available from the lead contact upon request. The ImageJ macro as well as the R code used to generate the filopodia maps were previously described and are available on GitHub (https://github.com/guijacquemet/FiloMAP).

## Supplemental information

Document S1. Figures S1–S6

Document S2. Article plus supplemental information
