# Supplementary material for: Myosin-X and talin modulate integrin activity at filopodia tips
Source: Cell Rep. 2021 Sep 14;36(11):109716. doi: 10.1016/j.celrep.2021.109716 (PMC8456781; doi:10.1016/j.celrep.2021.109716)
Supplement: Document S1. Figures S1–S6 [file mmc1.pdf]

Cell Reports, Volume *36*

Supplemental information

Myosin-X and talin modulate

integrin activity at filopodia tips

Mitro Miihkinen, Max L.B. Grönloh, Ana Popović, Helena Vihinen, Eija Jokitalo, Benjamin T. Goult, Johanna Ivaska, and Guillaume Jacquemet

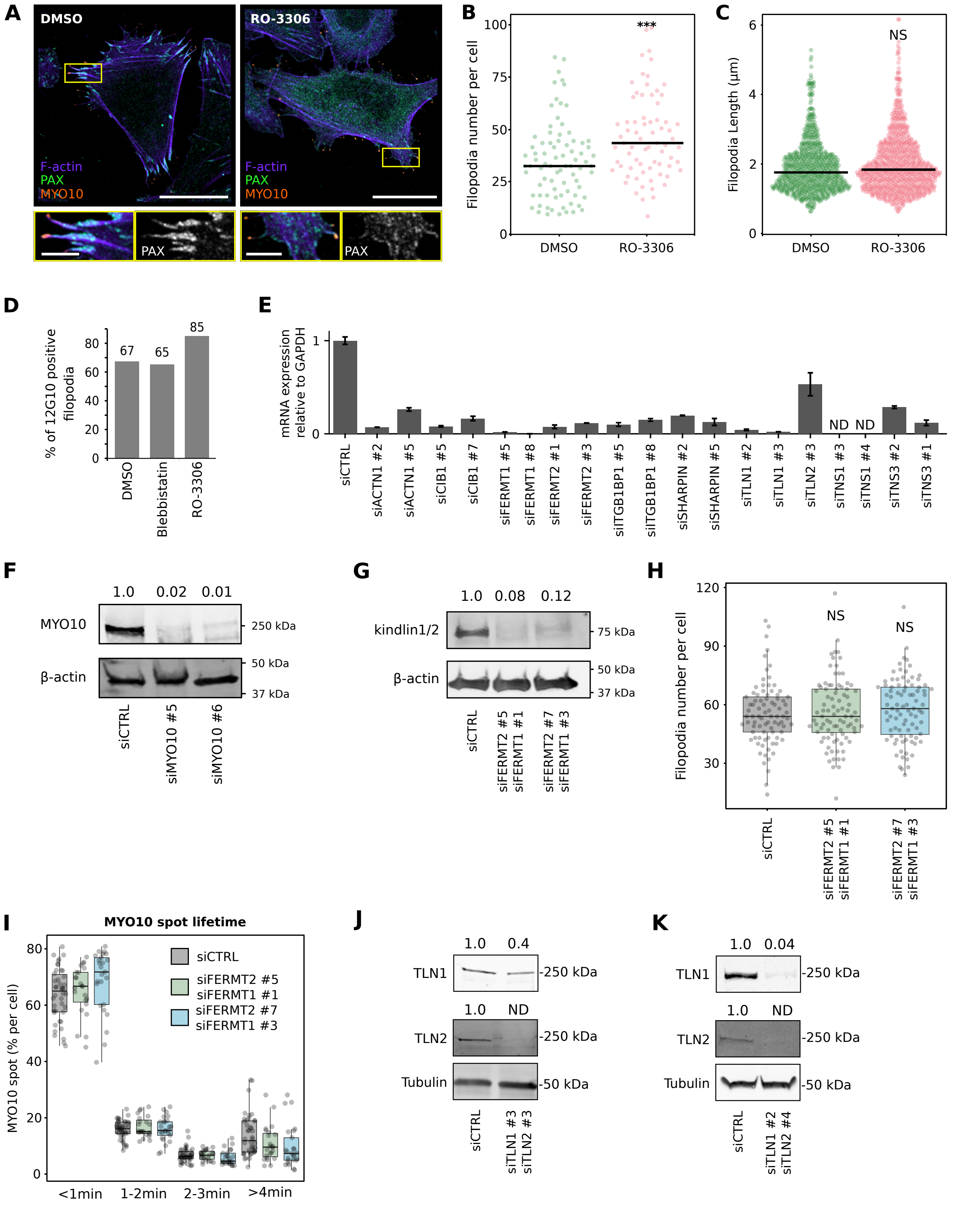

Fig. S1. Modulation of filopodia properties by focal adhesions and known integrin activity regulators, Related to Figure 1. (**A-C**) U2-OS cells expressing EGFP-MYO10 were plated on fibronectin for 1 h and treated for another hour with 10 μM RO-3306 (CDK1 inhibitor) or DMSO. Cells were stained for paxillin (PAX) and F-actin and imaged using an Airyscan confocal microscope or a spinning-disk confocal microscope. (**A**) Representative Airyscan images are displayed. The yellow rectangles highlight ROIs, which are magnified; scale bars: (main) 25 μm; (inset) 5 μm. (**B**) The number of MYO10-positive filopodia per cell was then quantified from the spinning-disk images (n > 72 cells, two biological repeats; *** p-value = 0.003). (**C**) Quantification of filopodia length, from SIM images, in U2-OS cells transiently expressing EGFP-MYO10 and treated for 1 h with 10 μM RO-3306 (CDK1 inhibitor) or DMSO (DMSO, n = 734 filopodia; RO-3306, n = 824 filopodia; three biological repeats; *** p-value = <0.001). (**D**) Bar chart highlighting the percentage of filopodia with detectable levels of active β1 integrin in cells treated with DMSO, blebbistatin or RO-3306 (DMSO, n = 734 filopodia; RO-3306, n = 824 filopodia; blebbistatin, n = 483 filopodia; three biological repeats). (**E**) The efficiency of siRNA-mediated silencing of each target of the siRNA screen performed in Fig 2A (except MYO10) was quantified by qPCR and normalized to GAPDH expression. The results were further normalized against expression detected in siCTRL cells.

Fig. S1. (Continued from previous page.) (**F**) Efficiency of siRNA-mediated silencing of MYO10 (oligos #5 and #6) in U2-OS cells validated by western blot. (**G**) Efficiency of dual siRNA-mediated silencing of FERMT1 and FERMT2 in U2-OS cells validated by western blot. (**H**) FERMT1- and FERMT2-silenced U2-OS cells transiently expressing EGFP-MYO10 were plated on fibronectin for 2 h, fixed, and the number of MYO10-positive filopodia per cell was quantified (n > 70 cells, three biological repeats). (**I**) FERMT1- and FERMT2-silenced U2-OS cells transiently expressing EGFP-MYO10 were plated on fibronectin and imaged live using an Airyscan confocal microscope (1 picture every 5 s over 20 min). For each condition, MYO10-positive particles were automatically tracked, and MYO10 spot lifetime (calculated as a percentage of the total number of filopodia generated per cell) was plotted and displayed as boxplots (see Methods for details; three biological repeats, more than 21 cells per condition). (**J-K**) Efficiency of dual siRNA-mediated silencing of TLN1 and 2 (oligos #3 and #3 or oligos #2 and #4) in U2-OS cells (one round of silencing) validated by western blot. For all panels, p-values were determined using a randomization test. NS indicates no statistical difference between the mean values of the highlighted condition and the control.

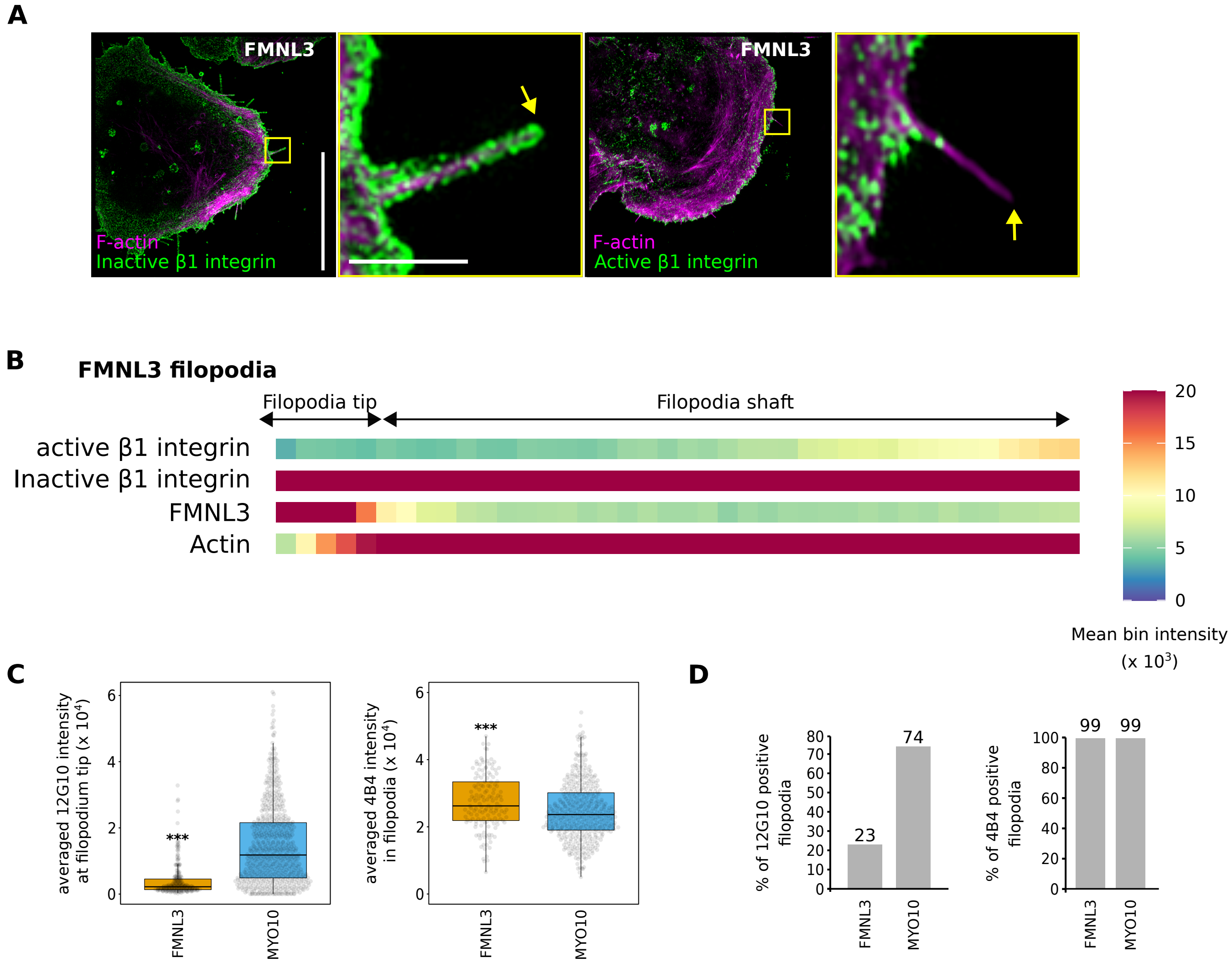

Fig. S2. FMNL3-induced filopodia lack active integrin at their tips, Related to Figure 3. (**A-C**) U2-OS cells expressing EGFP-FMNL3 were plated on fibronectin for 2 h, stained for active (antibody 12G10) or inactive (antibody mAb13) β1 integrin and F-actin, and imaged using SIM. (**A**) Representative MIPs are displayed. The yellow squares highlight ROIs, which are magnified; yellow arrows highlight filopodia tips; scale bars: (main) 20 µm; (inset) 2 µm. (**B**) Heatmap highlighting the sub-filopodial localisation of the proteins stained in (**A**) based on their intensity profiles (FMNL3, n = 373 filopodia; F-actin, n = 373 filopodia; active β1 integrin, n = 228 filopodia; inactive β1 integrin, n = 143 filopodia). (**C**) The average intensity of 12G10 at filopodia tips and of 4B4 in filopodia measured in (**B**) are displayed as box plots (*** p-value = <0.001). (**D**) Bar chart highlighting the percentage of FMNL3 and MYO10-induced filopodia with detectable levels of active (12G10) and inactive (4B4) β1 integrin (**C** and **D**; 12G10: FMNL3, n = 228 filopodia; MYO10, n = 329 filopodia; 4B4: FMNL3, n = 143 filopodia; MYO10, n = 413 filopodia). For all panels, p-values were determined using a randomization test.

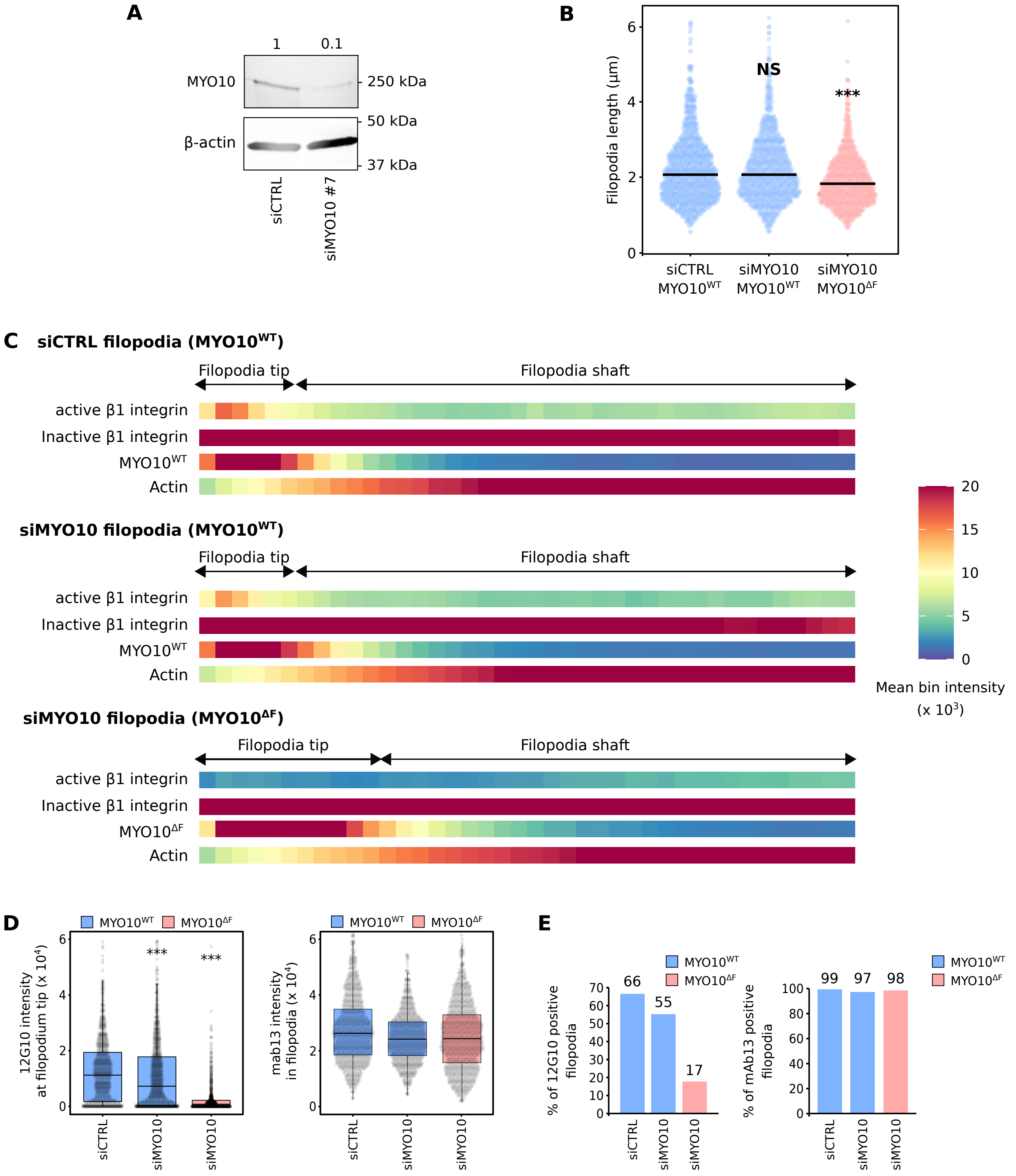

Fig. S3. MYO10-MyTH4/FERM is required for integrin activation but not localization in filopodia, Related to Figure 3. (**A**) The efficiency of siRNA-mediated silencing of MYO10 in U2-OS cells was validated by western blot. The siMYO10 #7 oligo targets the 3' UTR of the MYO10 mRNA. (**B-D**) MYO10-silenced U2-OS cells transiently expressing EGP-MYO10^{WT} or MYO10$^{\Delta F}$ were plated on fibronectin, stained for active (antibody 12G10) or inactive (antibody mAb13) β1 integrin and F-actin, and imaged using SIM. (**B**) Quantification of filopodia length, from the SIM images, are displayed as dotplots where the median is highlighted (siCTRL EGP-MYO10^{WT}, n = 799 filopodia; siMYO10 #7 EGP-MYO10^{WT}, n = 897 filopodia; siMYO10 #7 EGP-MYO10$^{\Delta F}$, n = 731 filopodia; three biological repeats; *** p value = <0.001). (**C**) Heatmap highlighting the sub-filopodial localisation of the indicated proteins based on their intensity profiles (siCTRL EGP-MYO10^{WT} filopodia: MYO10, n = 799 filopodia; F-actin, n = 799 filopodia; active β1 integrin, n = 799 filopodia; inactive β1 integrin, n = 878 filopodia. siMYO10 #7 EGP-MYO10^{WT} filopodia: MYO10, n = 897 filopodia; F-actin, n = 897 filopodia; active β1 integrin, n = 897 filopodia; inactive β1 integrin, n = 960 filopodia. siMYO10 #7 EGP-MYO10$^{\Delta F}$ filopodia: MYO10, n = 731 filopodia; F-actin, n = 731 filopodia; active β1 integrin, n = 731 filopodia; inactive β1 integrin, n = 778 filopodia. Three biological repeats). (**D**) The average intensity of 12G10 at filopodia tips and of mAb13 in filopodia measured in (**C**) are displayed as box plots (*** p-value = <0.001).

Fig. S3. (Continued from previous page.) (**E**) Bar chart highlighting the percentage of filopodia with detectable levels of active and inactive β1 integrin in the indicated conditions (**D** and **E**; active β1 integrin: siCTRL EGP-MYO10^{WT} filopodia, n = 799 filopodia; siMYO10 #7 EGP-MYO10^{WT}, n = 897 filopodia; siMYO10 #7 EGP-MYO10ΔF filopodia, n = 731 filopodia. Inactive β1 integrin: siCTRL EGP-MYO10^{WT} filopodia, n = 878 filopodia; siMYO10 #7 EGP-MYO10^{WT}, n = 960 filopodia; siMYO10 #7 EGP-MYO10$^{\Delta F}$ filopodia, n = 778 filopodia. Three biological repeats). For all panels, p-values were determined using a randomization test. NS indicates no statistical difference between the mean values of the highlighted condition and the control.

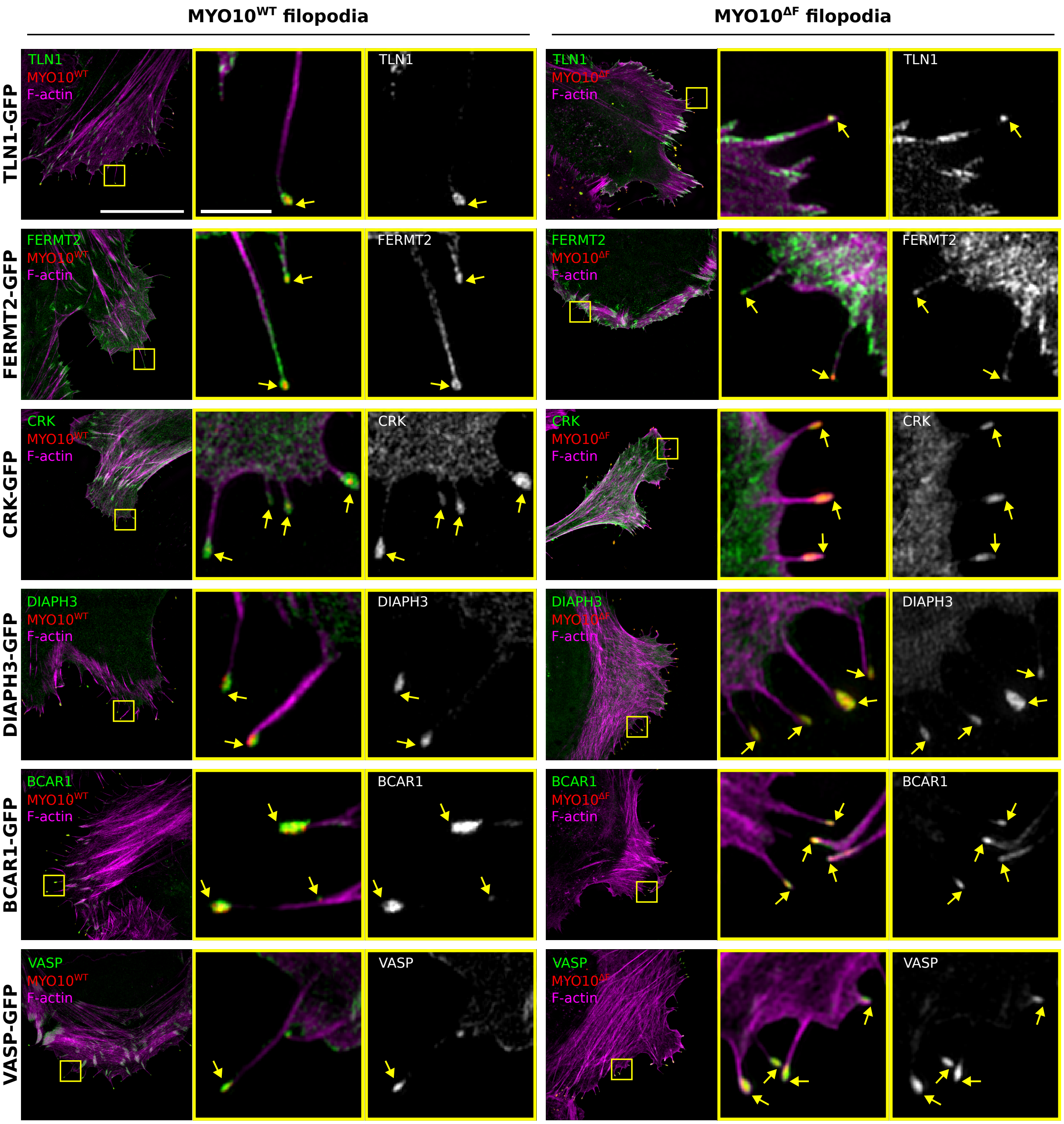

Fig. S4. MYO10-FERM deletion has minimal impact on known filopodia tip protein localization, Related to Figure 3. U2-OS cells expressing MYO10WT-mScarlet or MYO10ΔF-mScarlet-I together with TLN1-GFP, FERMT2-GFP, CRK-GFP, DIAPH3-GFP, BCAR1-GFP or VASP-GFP were plated on fibronectin for 2 h, fixed, stained for f-actin and imaged using SIM. Representative MIPs are displayed. The yellow squares highlight ROIs, which are magnified; yellow arrows highlight filopodia tips; scale bars: (main) 20 µm; (inset) 2 µm.

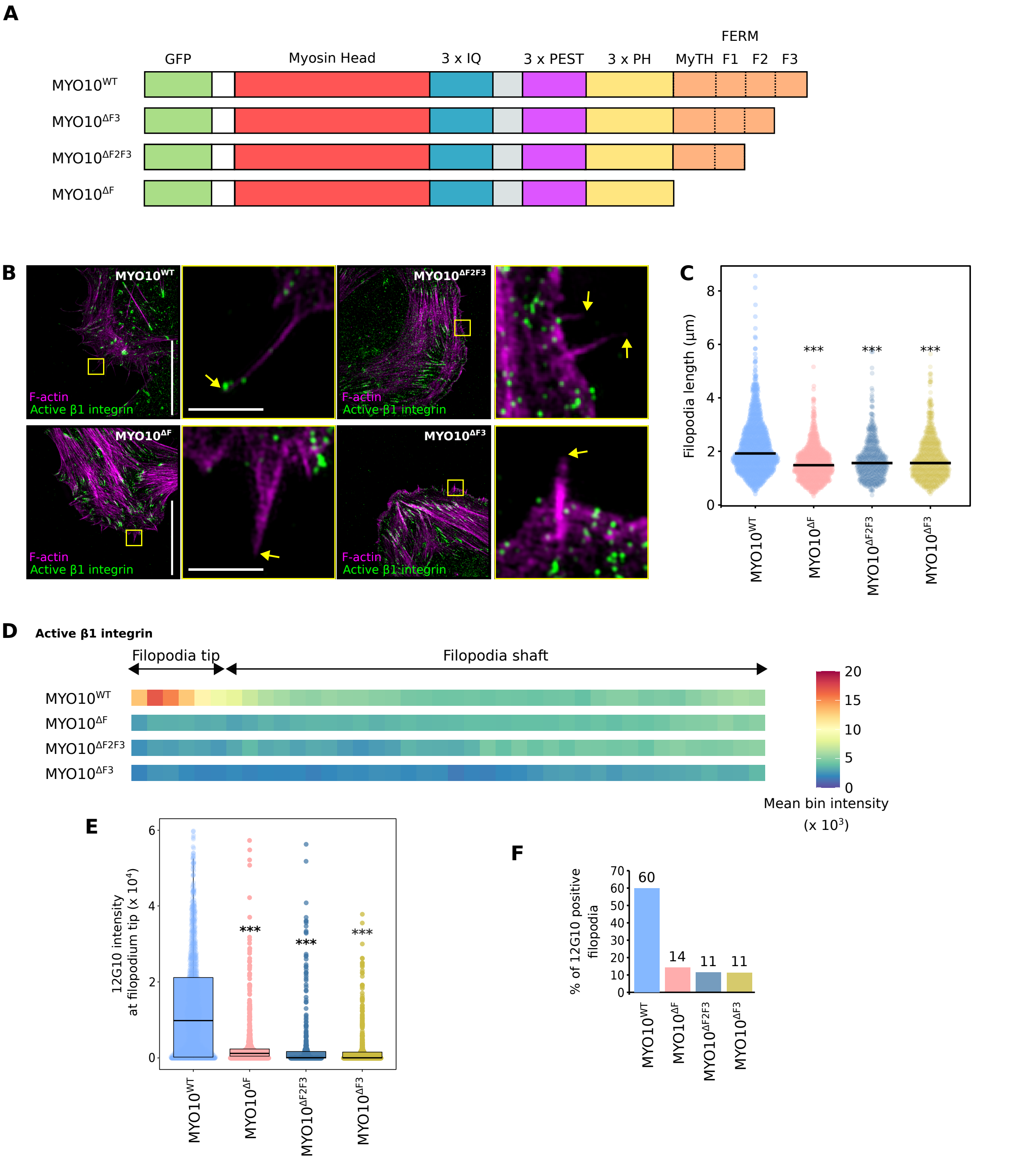

Fig. S5. The F3 subdomain of MYO10-FERM is required to activate integrins at filopodia tips, Related to Figure 4. (**A**) Cartoon illustrating the EGFP-MYO10WT, EGFP-MYO10ΔF, EGFP-MYO10$^{\Delta F2F3}$, and EGFP-MYO10$^{\Delta F3}$ constructs. (**B-E**) U2-OS cells transiently expressing EGFP-MYO10^{WT}, EGFP-MYO10$^{\Delta F}$, EGFP-MYO10$^{\Delta F2F3}$ or EGFP-MYO10$^{\Delta F3}$ were plated on fibronectin, stained for active β1 integrin (antibody 12G10) and F-actin, and imaged using SIM. (**B**) Representative MIPs are displayed. The yellow squares highlight ROIs, which are magnified; yellow arrows highlight filopodia tips; scale bars: (main) 20 µm; (inset) 2 µm. (**C**) Quantification of MYO10^{WT}, MYO10$^{\Delta F}$, MYO10$^{\Delta F2F3}$ and MYO10$^{\Delta F3}$ filopodia length, from the SIM images, are displayed as dot plots where the median is highlighted (*** p-value = <0.001). (**D**) Heatmap highlighting the sub-filopodial localization of active β1 integrin (antibody 12G10) in MYO10^{WT}, MYO10$^{\Delta F}$, MYO10$^{\Delta F2F3}$ and MYO10$^{\Delta F3}$ filopodia. (**E**) The average intensity of 12G10 at filopodia tips measured in (**D**) are displayed as box plots (*** p-value = <0.001). (**F**) Bar chart highlighting the percentage of MYO10^{WT}, MYO10$^{\Delta F}$, MYO10$^{\Delta F2F3}$ and MYO10$^{\Delta F3}$ filopodia with detectable levels of active β1 integrin. (**B-F**) EGP-MYO10^{WT}, n = 1073 filopodia; EGFP-MYO10$^{\Delta F}$, n = 776 filopodia; MYO10$^{\Delta F2F3}$, n = 497 filopodia; MYO10$^{\Delta F3}$, n = 723 filopodia; Three biological repeats. For all panels, p-values were determined using a randomization test.

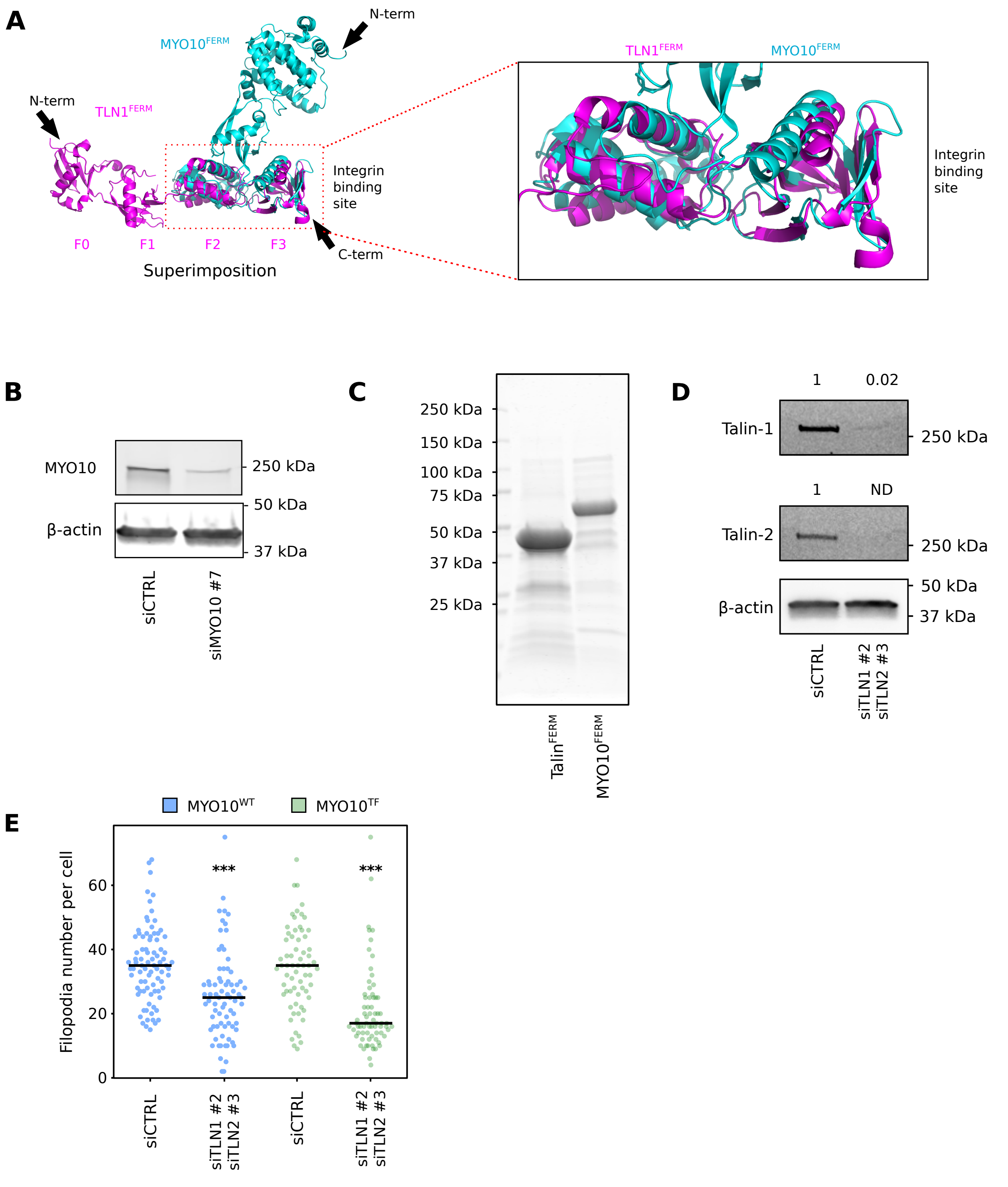

Fig. S6. MYO10-FERM and talin-FERM structure and function in filopodia, Related to Figure 6. (**A**) Visualisation of the structure of MYO10-FERM (PDB: 3PZD) and TLN1-FERM (PDB: 3IVF) domains using PyMOL. The black arrows indicate the protein orientation from N to C terminal. The two FERM domains were superimposed to highlight their structural homology and differences. The integrin-binding region on the talin-FERM domain is highlighted and magnified. (**B**) The efficiency of siRNA-mediated silencing of MYO10 in MDA-231 cells was validated by western blot. The siMYO10 #7 oligo targets the 3' UTR of the MYO10 mRNA. (**C**) Recombinant his-tagged TLN1 and MYO10-FERM domains were produced in bacteria and subsequently purified using a gravity Ni2+-column. A representative gel stained with Instant blue is displayed. (**D**) Efficiency of dual siRNA-mediated silencing of TLN1 and TLN2 in U2-OS cells following two rounds of silencing. A representative western blot is displayed. (**E**) TLN1 and TLN2-silenced U2-OS cells transiently expressing EGFP-MYO10^{WT} or EGFP-MYO10^{TF} were plated on fibronectin for 2 h, fixed, stained, imaged using a spinning-disk confocal microscope, and the number of MYO10-positive filopodia per cell was quantified (siCTRL/MYO10^{WT}, n = 83 cells; siCTRL/MYO10^{TF}, n = 66 cells; siTLN1 and siTLN2/MYO10^{WT}, n = 75 cells; siTLN/MYO10^{TF}, n = 71 cells; three biological repeats, *** p-value < 0.001). For all panels, p-values were determined using a randomization test.
